# LAFITE Reveals the Complexity of Transcript Isoforms in Subcellular Fractions

**DOI:** 10.1002/advs.202203480

**Published:** 2022-12-03

**Authors:** Jizhou Zhang, Xiao Lin, Yuelong Chen, Tsz‐Ho Li, Alan Chun‐Kit Lee, Eugene Yui‐Ching Chow, William Chi‐Shing Cho, Ting‐Fung Chan

**Affiliations:** ^1^ School of Life Sciences The Chinese University of Hong Kong Shatin Hong Kong SAR China; ^2^ State Key Laboratory of Agrobiotechnology The Chinese University of Hong Kong Shatin Hong Kong SAR China; ^3^ Department of Clinical Oncology Queen Elizabeth Hospital Kowloon Hong Kong SAR China

**Keywords:** direct RNA‐sequencing, full‐length transcripts, long read, nanopore, subcellular fraction

## Abstract

Characterization of the subcellular distribution of RNA is essential for understanding the molecular basis of biological processes. Here, the subcellular nanopore direct RNA‐sequencing (DRS) of four lung cancer cell lines (A549, H1975, H358, and HCC4006) is performed, coupled with a computational pipeline, **L**ow‐abundance **A**ware **F**ull‐length **I**soform clus**TE**r (LAFITE), to comprehensively analyze the full‐length cytoplasmic and nuclear transcriptome. Using additional DRS and orthogonal data sets, it is shown that LAFITE outperforms current methods for detecting full‐length transcripts, particularly for low‐abundance isoforms that are usually overlooked due to poor read coverage. Experimental validation of six novel isoforms exclusively identified by LAFITE further confirms the reliability of this pipeline. By applying LAFITE to subcellular DRS data, the complexity of the nuclear transcriptome is revealed in terms of isoform diversity, 3'‐UTR usage, m6A modification patterns, and intron retention. Overall, LAFITE provides enhanced full‐length isoform identification and enables a high‐resolution view of the RNA landscape at the isoform level.

## Graphical abstract

Characterization of the subcellular distribution of RNA is essential for understanding the molecular basis of biological processes. By performing a subcellular nanopore direct RNA sequencing, coupled with a full‐length isoform detection pipeline, LAFITE, the authors highlight the divergence between cytoplasmic and nuclear transcriptome, in terms of isoform diversity, 3'‐UTR usage, m6A modification patterns, and alternative splicing.

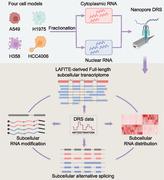

## 1 Introduction

The asymmetric distribution of RNA molecules over a significant proportion of the transcriptome has been reported in numerous organisms, from unicellular to multicellular organisms.^[^ ^1^, ^2^ ^]^ This widely appreciated phenomenon has emerged as an essential program of post‐transcriptional regulation that efficiently governs the accumulation of protein products in specified cellular domains,^[^ ^3^ ^]^ and thereby determines the architecture of cellular components and fundamental biological processes.^[^ ^4^, ^5^ ^]^ Aberrant RNA subcellular localization has been implicated in the pathogenesis of many diseases, including Huntington's disease,^[^ ^6^ ^]^ amyotrophic lateral sclerosis,^[^ ^7^ ^]^ and cancer.^[^ ^8^, ^9^ ^]^ Therefore, a systematic elucidation of the subcellular fate of RNA molecules has the potential to reveal the underlying mechanisms that regulate tissue development and disease progression.

Advances in technology have accelerated the revolution of molecular approaches to monitor the subcellular RNA localization, but few of them can achieve resolution on a transcriptome‐wide scale.^[^ ^10^, ^11^ ^]^ The most classic and robust methods are image‐based fluorescence in situ hybridization (FISH) and RNA beacon techniques, which are designed to directly visualize and quantify the subcellular distribution of RNA using fluorophore‐labeled oligonucleotides.^[^ ^12^, ^13^ ^]^ However, the requirement for predesigned probes restricts their application to well‐characterized transcripts, thereby hindering the analysis of novel transcripts. In addition, nonspecific hybridization has been shown to cause major disruptions to the RNA localization signal, particularly for transcripts with repeat sequences.^[^ ^14^ ^]^ Above all, the large burden imposed by the need to generate transcriptome‐wide probes makes these techniques inaccessible to most.

The recent advent of next‐generation sequencing (NGS) has provided an alternative approach to investigate the subcellular distribution of various RNA species, including mRNA, long non‐coding RNA (lncRNA), and circular RNA, by sequencing RNA molecules isolated from different cellular compartments.^[^ ^15^ ^–^ ^20^ ^]^ This type of fractionation sequencing, and its subsequent revisions, have greatly improved the sensitivity and scale of profiling intercellular RNA localization.^[^ ^21^, ^22^ ^]^ However, the short read length of NGS is a major drawback, as it limits the characterization of transcriptome diversity due to alternative splicing (AS) and alternative transcription start sites and termination sites.^[^ ^23^, ^24^ ^]^ As a result, studies of the subcellular transcriptome have mainly focused on the gene level, and isoform‐level characterization remains relatively rare. Different isoforms encoded by the same gene may have divergent functions and subcellular localizations.^[^ ^25^ ^]^ Additionally, the fragmentation‐then‐assembly strategy impedes the reconstruction of full‐length transcripts,^[^ ^26^ ^]^ leading to the considerable omission of transcript elements (e.g., untranslated regions [UTRs]).^[^ ^27^ ^]^ There is accumulating evidence that *cis*‐regulatory elements in the UTR may affect RNA transportation and localization.^[^ ^28^, ^29^ ^]^ Therefore, a comprehensive analysis of the subcellular fate of RNA molecules requires knowledge of the full‐length subcellular transcriptome.

Nanopore direct RNA‐sequencing (DRS) has emerged as a promising technique to capture ultra‐long RNA molecules. It has been widely used to resolve full‐length transcripts in eukaryote transcriptomes.^[^ ^30^, ^31^ ^]^ Thus, DRS is well suited to deciphering the complex transcriptome, particularly the nuclear transcriptome, which often comprises very long transcripts with intron retentions.^[^ ^32^ ^]^ Moreover, DRS advances in profiling native sequence signals without requiring cDNA conversion, which further promises the detection and quantification of RNA modifications.^[^ ^33^ ^]^ Such dynamic and extensive RNA modifications appear to be critical regulators of the subcellular localization of transcripts.^[^ ^34^, ^35^ ^]^ Nonetheless, subcellular transcriptomic analysis using DRS technology has not yet been reported.

Challenges still exist in teasing out full‐length transcripts from DRS data. In addition to the predicament that some tools, including ToFu^[^ ^36^ ^]^ and SQANTI,^[^ ^37^ ^]^ were primarily developed for use with PacBio Iso‐Seq technology, the remaining tools available for isoform detection from DRS data, such as FLAIR,^[^ ^38^ ^]^ StringTie,^[^ ^39^ ^]^ and TrackCluster,^[^ ^40^ ^]^ mainly build upon sequence coverage to filter potential assembly noise, leading to the omission of transcripts with few reads support. Importantly, such low‐abundance transcripts have been found to be extensively expressed in various tissues^[^ ^41^ ^]^ and function as pilot regulators in metabolic programs^[^ ^42^ ^]^ and cancer progression.^[^ ^43^, ^44^ ^]^ Hence, a new tool that is more sensitive to low‐abundance transcripts is urgently needed.

To address the abovementioned issues, we performed subcellular fractionation of four lung cancer cell lines (A549, H1975, H358, and HCC4006), followed by Nanopore DRS to profile the native RNA molecules in the cytoplasmic and nuclear fractions. A computational pipeline, **L**ow‐abundance **A**ware **F**ull‐length **I**soform clus**TE**r (LAFITE) was further developed to define high‐consensus, full‐length isoforms and rescue the isoforms with low read coverage. Performance evaluation using DRS data from synthetic spike‐in RNA variants (SIRV) and real samples and other orthogonal datasets indicated high precision and integrity of the isoforms identified by LAFITE. Moreover, using LAFITE, we found extensive divergence in isoform diversity, alternative UTR usage, AS, and RNA modifications between the cytoplasm and nucleus. Taken together, our findings provide the first profile of the full‐length subcellular transcriptome, which will be a valuable resource for interpreting the underlying biological functions of RNA molecules. Furthermore, the utility and accuracy of LAFITE allow the easy use of long‐read RNA sequencing (RNA‐seq) data to gain insights into the complexity of the transcriptome.

## 2 Results

### 2.1 Generation of Full‐Length Transcriptomes of Subcellular Fractions

**Figure 1:**
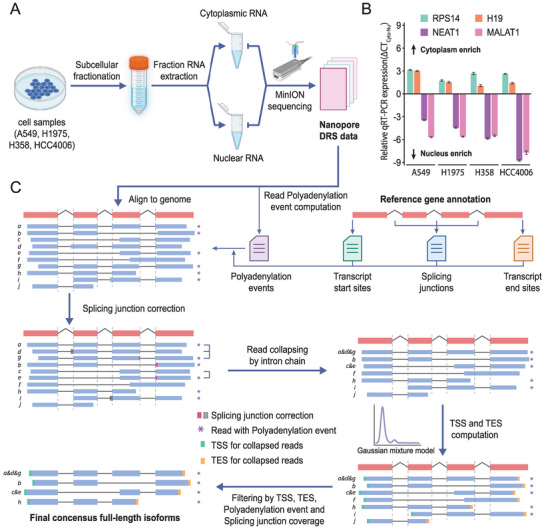
LAFITE generated a full‐length subcellular transcriptome from fractionation Nanopore DRS data. A) Experimental design to generate Nanopore direct RNA‐sequencing (DRS) data for the cytoplasmic and nuclear fractions from four model cell lines (A549, H1975, H358, and HCC4006). Created with BioRender.com. B) Validation of the subcellular fractionation results using quantitative real‐time polymerase chain reaction (qRT‐PCR) analyses of cytoplasmic (*H19*, *RPLS14*) and nuclear marker genes (*MALAT1*, *NEAT1*). The expression of the marker genes in each fraction was measured in three technical replicates. The relative cytoplasm‐to‐nucleus expression of the marker genes was indicated by mean CT_cytoplasm_‐CT_nucleus_ (abbreviated as ΔCT_Cyto‐Nu_). The error bars represent the standard deviation. C) Overview of the Low‐abundance Aware Full‐length Isoform clusTEr (LAFITE) pipeline to identify high‐consensus full‐length isoforms from DRS data. First, LAFITE takes the alignment file as the input with the supplementary reference gene annotation and read polyadenylation results. Next, a reference‐based splicing junction error correction is performed to correct all novel and low‐confidence splicing sites in the reads. The corrected reads are then grouped and collapsed according to their intron chain (splicing structure). Subsequently, LAFITE employs the Gaussian mixture model to compute the transcript start site (TSS) and transcript end site (TES) for the collapsed reads. Finally, a multistep assessment is implemented to remove potential artifacts and truncated isoforms.

To systematically identify full‐length transcripts in both nuclear and cytoplasmic fractions, we performed subcellular fractionation followed by Nanopore DRS on four lung adenocarcinoma cell lines (A549, H1975, H358, and HCC4006; **Figure** **1**A). Quality control assessment of the fractionation purity using quantitative real‐time polymerase chain reaction (qRT‐PCR) based on selected cytoplasmic (*H19*, *RPLS14*) and nuclear (*MALAT1*, *NEAT1*) marker genes demonstrated the effective separation of the subcellular fractions in the four cell samples (Figure 1B). Follow‐up sequencing yielded more than 13 million long reads, with a high percentage of reads passing quality control. The average read length was 700–1,400 bp, which was better than that from the previous study,^[^ ^30^ ^]^ indicating that the input RNA molecules were of high quality (Table S1, Supporting Information). We also performed standard Illumina RNA‐seq of each fraction for relevant assessments. Further gene expression quantification based on the DRS and Illumina RNA‐seq data also revealed the consistent enrichment of marker genes in the corresponding subcellular compartments (Figure S1, Supporting Information), thus confirming the efficient fractionation and reproducibility across different cell lines.

The reads that passed quality filtering were then subjected to full‐length transcriptome assembly. Isoform detection from long reads is primarily based on the same strategy used for short‐read transcriptome assembly, which relies heavily on sequence coverage. However, due to the throughput limitations of Nanopore DRS, low‐abundance isoforms have an extremely high chance of being detected with only a few reads. In silico simulations suggested that transcripts with an expression level of 1 transcript per million (TPM) had more than a 90% probability of having two or fewer reads in a DRS sequencing run with a total throughput of 1 M reads (Figure S2, Supporting Information). Such transcripts are easily overlooked using existing state‐of‐the‐art tools, such as FLAIR, StringTie, and TrackCluster. Given the ability of long reads to resolve complex transcript structures in single molecules, the main obstacles to isoform calling are the artifacts and truncated reads that are introduced due to the high sequencing error rate, RNA fragmentation, and pore blocking.^[^ ^45^ ^]^ Thus, we developed the LAFITE pipeline to explicitly identify high‐consensus isoforms and retrieve low‐abundance isoforms from Nanopore DRS data (Figure 1C).

LAFITE starts with DRS data in BAM format, using the reference gene annotation as the guide for splicing junction error correction. By default, LAFITE only corrects the reported splicing site with an annotated splicing site if the edit distance is within 40 bp. The corrected reads are then collapsed based on their intron chain. Next, LAFITE models a Gaussian mixture distribution to estimate the putative transcript start site (TSS) and transcript end site (TES) for each collapsed read, based on the start and end sites from all reads within the cluster. Finally, a three‐step filtering process is implemented to remove potential artifacts and truncated reads with disqualified splicing structures, TSSs, and TESs. In the first step, novel splicing junctions (either donor or acceptor sites) with fewer than two DRS reads and their parent collapsed reads are discarded. In the second step, novel collapsed reads without sufficient polyadenylation events from the raw reads are excluded. Nanopore DRS is designed to sequence poly(A)+ RNA; thus, reads with polyadenylation events are a great indicator of a completed sequencing process. In the third step, collapsed reads with TSSs that are not supported by annotated TSS or 5′ cap analysis gene expression (CAGE) data are removed. The remaining collapsed reads are considered as high‐consensus, full‐length transcripts.

### 2.2 LAFITE Outperforms Existing Tools in Identifying Full‐Length Isoforms

**Figure 2:**
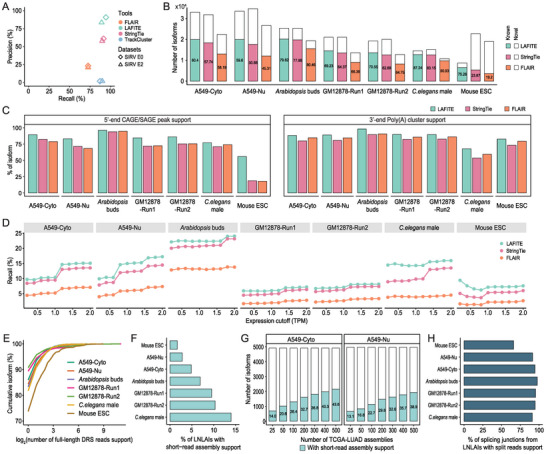
Evaluation of LAFITE performance. A) Comparison of assembly accuracy (precision and recall) of LAFITE, FLAIR, StringTie, and TrackCluster on two synthetic spike‐in RNA variant datasets. B) Stacked bar plot depicting the performance of LAFITE, FLAIR, and StringTie with seven DRS datasets derived from real transcriptomes. The y‐axis represents the number of the isoforms identified by different tools in different datasets. The color bars represent the known isoforms detected. The number in the bar represents the precision as indicated by the proportion of known isoforms among all identified isoforms. C) The proportion of the TSSs of the assembled isoforms in the seven DRS datasets supported by 5′ cap analysis gene expression (CAGE)/serial analysis gene expression (SAGE) peaks, or TESs supported by the presence of 3′ poly(A) clusters. D) Recall of the assembled isoforms among all expressed known isoforms (transcripts per million [TPM] > 0) under different expression cutoff values. Isoform expression was quantified using Nanocount with the DRS data. E) Cumulative distribution curve showing the number of reads for the novel isoforms uniquely identified by LAFITE in the seven datasets. The x‐axis corresponds to the isoforms with different numbers of full‐length DRS reads, and the y‐axis corresponds to the cumulative fraction of the novel isoforms. F) Bar plot showing the percentage of LAFITE‐exclusive novel low‐abundance isoforms (LNLAIs) obtained with assembly support from the Illumina RNA‐seq datasets. G) Bar plot showing a steady increase in the number of LNLAIs with assembly support as the number of datasets from The Cancer Genome Atlas Lung Adenocarcinoma (TCGA‐LUAD) cohort increases. H) Bar plot showing the percentage of the splicing junctions from LNLAIs matched to the split reads from Illumina RNA‐seq datasets.

To conduct a comprehensive assessment, we initially benchmarked the performance of LAFITE against FLAIR, StringTie, and TrackCluster using DRS datasets from the SIRV control mix (Lexogen, Vienna, Austria). SIRV RNAs are designed to mimic the complexity of human transcripts. They consist of 69 synthetic isoforms derived from seven gene loci. This ground truth enables the calculation of standard performance metrics, such as precision and recall. We defined precision and recall as the fraction of correctly identified isoforms out of all assembled isoforms and the reference isoforms, respectively. Consequently, LAFITE showed similar or greater recall than the other tools and achieved the highest precision of all the tools tested (**Figure** **2**A). This result demonstrated a marked improvement of LAFITE in minimizing the assembly noise while preserving nearly the same number of true isoforms when compared with the other tools, thereby reducing the time and effort required for subsequent validation experiments.

Although they are modeled on human genes, SIRV isoforms are still unable to capture all of the characteristics of a genuine transcriptome. Hence, we further examined the performance of the assemblers using seven DRS datasets derived from real transcriptomes, including three from *Arabidopsis thaliana* buds (*Arabidopsis* buds), *Caenorhabditis elegans* males (*C. elegans* males), and mouse embryonic stem cells (mouse ESC); two from human GM12878 cell line (GM12878‐Run1, GM12878‐Run2); and two from our study (A549 cytoplasm [A549‐Cyto] and A549 nucleus [A549‐Nu]). All tools were supplied with the reference gene annotation from either GENCODE (for human and mouse) or Ensembl (for *Arabidopsis* and *C. elegans*). TrackCluster was excluded from the comparison due to its excessively slow speed (more than 200 times slower than LAFITE) and markedly high false‐positive rate for SIRV data. It should be noted that ground truth information is not available for real transcriptomes, as we cannot be certain which isoforms are genuinely present in the dataset. Nonetheless, it is standard practice to consider the isoforms in a reference annotation as the ground truth set and implement the evaluation following the aforementioned criteria.^[^ ^39^, ^46^ ^]^ In our assessment, LAFITE consistently outperformed FLAIR and StringTie, in particular, and achieved the highest recall with more annotated isoforms reported across all seven datasets. We observed increased precision for FLAIR on *Arabidopsis* buds and *C. elegans* males; however, this came at the cost of a steep decrease in recall (Figure 2B, Figure S3, Supporting Information). This is the general trade‐off between precision and recall, especially for post‐assembly filtering approaches merely based on coverage. LAFITE was orchestrated to balance this trade‐off, and thus it achieved better performance than all of the alternative tools.

Additionally, we employed orthogonal datasets to investigate the integrity of the isoforms detected using the three tools, including the TSS peak from CAGE or serial analysis gene expression (SAGE) data and 3′ polyadenylation site data. 5′‐end CAGE/SAGE and 3′‐end sequencing are effective techniques to characterize TSSs and TESs at single‐nucleotide resolution.^[^ ^47^ ^–^ ^49^ ^]^ Notably, we found that the TSSs and TESs of the isoforms detected by LAFITE had the greatest overlap with the CAGE/SAGE peaks and poly(A) clusters, respectively (Figure 2C). Consistently high supporting rates could also be observed for the TSSs and TESs from the novel and low‐abundance isoforms (Figure S4, Supporting Information). Collectively, these evaluations confirmed the higher quality of the full‐length isoforms identified by LAFITE than those identified using the other tools.

Previous studies have described significant deviations in gene annotations from different consortia,^[^ ^50^, ^51^ ^]^ implying that the selection of a reference standard may affect the performance metrics to a certain degree. Nevertheless, we still observed excellent performance of LAFITE when using the reference annotation from RefSeq (Figure S5, Supporting Information). Meanwhile, we noticed an unexpectedly low number of isoforms reported by LAFITE in the mouse ESC dataset, at less than half the number reported by StringTie and FLAIR (Figure 2B, Table S2, Supporting Information). A comparison to the reference annotation using GffCompare^[^ ^52^ ^]^ showed that most of isoforms assembled by StringTie and FLAIR (49% and 64%) were classified as “contained in reference” (Table S2, Supporting Information). Moreover, we found that more than 75% of isoforms assembled by StringTie and FLAIR lacked support from CAGE peaks for the TSSs (Figure 2C). Importantly, an inspection of read coverage along the gene body revealed a strong bias toward the 3′‐end in the mouse ESC DRS data (Figure S6, Supporting Information). This concordance may reflect substantially truncated isoforms identified by StringTie and FLAIR due to degradation of the input RNA. By contrast, LAFITE exhibited a high level of robustness at identifying full‐length isoforms and eliminating truncated isoforms, even for low‐quality data.

### 2.3 LAFITE Enables Improved Detection of Low‐Abundance Isoform

Subsequently, we investigated the assemblers' performance at detecting low‐abundance isoforms. To this end, we proposed a similar metric by examining the fraction of assembled isoforms among all expressed reference isoforms under different expression cutoffs. The abundance of the reference annotated isoforms in each dataset was quantified using NanoCount.^[^ ^53^ ^]^ Isoforms were considered for the test if their expression levels were lower than the cutoff. Remarkably, LAFITE consistently obtained the highest recall of the tested tools under all RNA expression thresholds, especially for isoforms with expression levels of 1 TPM or less (Figure 2D). This pronounced recall demonstrated the exceptional sensitivity of LAFITE at identifying isoforms with low expression levels in DRS data. We further surveyed the novel isoforms uniquely identified by LAFITE and found that the overwhelming majority (73‐91% depending on the dataset) had two or fewer full‐length DRS reads support (Figure 2E), which confirmed the omission of low‐abundance isoforms when using existing methods.

To validate the existence of these LAFITE‐exclusive novel low‐abundance isoforms (LNLAIs), we used publicly available Illumina short‐read RNA‐seq data corresponding to each tissue/cell line (except A549‐Cyto and A549‐Nu, for which we used in‐house sequencing data) to examine the supporting evidence for the isoform structure. An isoform was considered to have short‐read assembly support if it was assigned a class code of “=” after comparing it with short‐read assemblies using GffCompare.^[^ ^52^ ^]^ Despite the batch effect and sample variance between the DRS and Illumina data, we observed that a considerable proportion of LNLAIs received assembly support from short‐read assemblies, with the exception of mouse ESCs, possibly due to dynamic changes in transcriptome composition during cell differentiation^[^ ^54^ ^]^ (Figure 2F). Taking advantage of a large number of RNA‐seq datasets in The Cancer Genome Atlas (TCGA), we further inspected the supporting evidence for the LNLAIs from A549‐Cyto and A549‐Nu datasets using 538 tumor datasets from TCGA Lung Adenocarcinoma (TCGA‐LUAD) cohort. Consequently, we observed a steady increase in assembly support for LNLAIs as the sample size increased (Figure 2G). However, the intrinsic defect in read length notoriously limits the capacity of short reads to fully reconstruct the transcript isoform, particularly for transcripts with low levels of expression, which typically receive insufficient sequencing coverage.^[^ ^55^ ^]^ Splicing‐junction‐level support may thus provide reliable evidence for LNLAIs. Indeed, we observed a remarkably high support rate for the splicing junctions from LNLAIs by matching them to the split reads from Illumina RNA‐seq datasets, suggesting most splicing junctions from LNLAIs are accurate to single‐nucleotide level (Figure 2H). Collectively, these results emphasized the reliability of LAFITE in capturing low‐abundance transcripts.

### 2.4 Validation of the Novel Low‐Abundance Isoforms Identified by LAFITE

**Figure 3:**
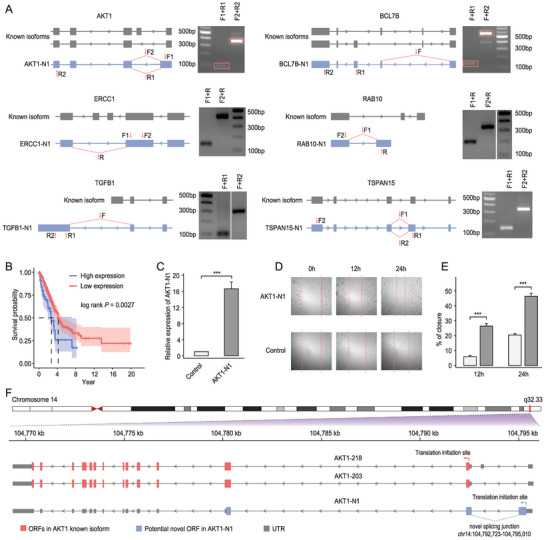
Functional validation of low‐abundance novel isoforms uniquely identified by LAFITE. A) PCR confirmed the existence of the novel isoforms. The schematic diagram shows the difference in splicing between the reference and novel isoforms. Primers designed for PCR validation are marked in red. All primer pairs were designed to span the specific splicing junction of the novel isoforms. The product size of each primer pair is indicated. Target bands for *AKT1‐N1* and *BCL7B‐N1* are enclosed by red rectangle. B) Kaplan–Meier curves of 503 LUAD patients in TCGA‐LUAD cohorts with high and low levels of *AKT1‐N1* expression (*p* = 0.0027, Log‐rank test). C) Relative expression of *AKT1‐N1* in A549 cells transfected with plasmid or control vectors. The expression of *AKT1‐N1* was normalized to the expression of *GAPDH*. Each target was measured by qRT‐PCR in three biological replicates and three technical replicates. The error bars represent the standard deviation. (*** *p* < 0.001, two‐sided Student's t‐test). D,E) Scratch wound‐healing assay showing the effect of *AKT1‐N1* overexpression on the motility of A549 cells (*** *p* < 0.001, two‐sided Student's t‐test). F) Schematic diagram showing the structure of *AKT1‐N1* and the known isoforms of *AKT1*. A novel ORF (colored in blue) nearest to 5′‐end was found in *AKT1‐N1*.

To experimentally verify the existence of the novel isoforms, six LNLAIs (*AKT1*‐*N1*, *BCL7B‐N1, ERCC1*‐*N1*, *RAB10*‐*N1*, *TGFB1‐N1, TSPAN15‐N1*) from well‐studied genes (*AKT1*, *BCL7B, ERCC1*, *RAB10*, *TGFB1, TSPAN15*) identified in the A549‐Cyto dataset were selected and validated using PCR. All six novel isoforms were detectable with different sets of primer pairs that were designed to span specific splicing junctions (**Figure** **3**A). The sequences of the PCR products were subsequently confirmed by Sanger sequencing (File S1, Supporting Information).

To investigate the biological impact of these LNLAIs, we first estimated the Kaplan–Meier curves for the survival time of the patients in the TCGA‐LUAD cohort in response to the expression of candidate LNLAIs. Notably, we observed a significant negative correlation between the expression of *AKT1‐N1* and patients’ overall survival (Figure 3B), suggesting a potential oncogenic function of *AKT1‐N1*. *AKT1* is a key regulator of signaling pathways involved in cell survival, migration, and growth. A growing body of evidence has confirmed a tumor‐inhibitor function of *AKT1* in multiple cancers by suppressing cell migration and invasion.^[^ ^56^ ^–^ ^58^ ^]^ This antithetical result thereby drives us to study the potential function of *AKT1‐N1*.

We next ectopically expressed *AKT1‐N1* in A549 cells by cloning and transient transfection. A wound‐healing assay was then performed to examine cell migration. qRT‐PCR results indicated the successful overexpression of *AKT1‐N1* in A549 cells (Figure 3C), and the forced expression of *AKT1‐N1* significantly promoted cell migration (Figure 3D,E). We further surveyed the open reading frame (ORF) harbored in *AKT1‐N1* and found a novel intact ORF nearest the 5′‐end (Figure 3F). According to the first‐AUG rule for mRNA translation,^[^ ^59^ ^]^ *AKT1‐N1* may have a high potential to encode a new protein in 135 amino acids. *In silico* function prediction using a convolutional neural network‐based method, DeepFRI,^[^ ^60^ ^]^ showed that this candidate protein is significantly associated with several metabolic processes (Table S3, Supporting Information), implying that *AKT1‐N1* might deliver its oncogenic function by encoding a novel protein to disturb the cellular metabolic processes. These results confirmed the presence and functional importance of low‐abundance isoforms identified by LAFITE. Taken together, these data shows that LAFITE has superior performance to current state‐of‐the‐art tools for full‐length isoform identification, particularly for low‐abundance isoforms.

### 2.5 Full‐Length Subcellular Transcriptome Profile

**Figure 4:**
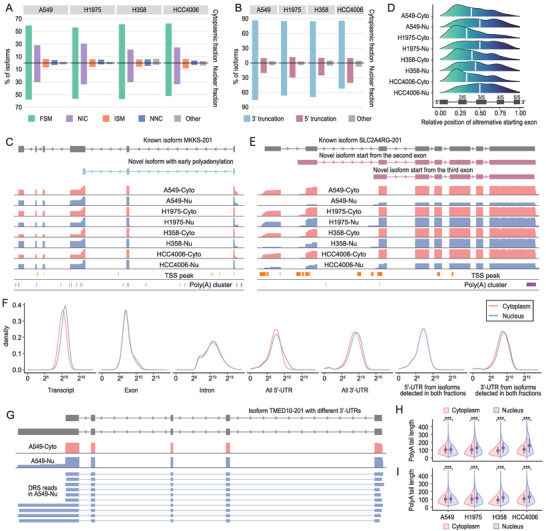
Overview of the full‐length subcellular transcriptome. A) Classification of the isoforms assembled in each fraction, colored by isoform structural categories from Figure S7. FSM: Full‐splice Match, ISM: Incomplete Splice Match, NIC: Novel In Catalog, NNC: Novel Not in Catalog. B) Classification of the ISMs identified in each fraction. C) Schematic diagram and genomic coverage plot showing a novel isoform from the *MKKS* gene derived from an early polyadenylation event, with 3ʹ poly(A) cluster supporting the previously unknown TES. D) Position of the start exon of the ISM classified as 5′ truncation in relation to the exon in the reference parent transcript, calculated by dividing the start exon position by the total number of exons in the reference parent transcript. E) Schematic diagram and genomic coverage plot illustrating two novel isoforms from the *SLC2A4RG* gene with alternative starting exons, with 5ʹ CAGE peaks support for the alternative TSSs. F) Density plot showing the length distribution of the transcript, exon, intron, 5ʹ‐UTR, and 3ʹ‐UTR detected in H358 cytoplasmic and nuclear fractions. G) Schematic diagram and genomic coverage plot demonstrating the alternative 3′‐UTR of isoform *TMED‐201* detected in the nuclear fraction. H) Length distribution of the poly(A) tail detected in the cytoplasmic and nuclear fractions (***p < 0.001, Mann‐Whitney U test). The poly(A) tail length for each read was estimated by nanopolish based on the raw signal. I) Length distribution of the poly(A) tails for the isoforms detected in both fractions (***p < 0.001, Mann‐Whitney U test).

We next applied LAFITE to our fractionation DRS data to interrogate the isoform profile at the subcellular level. On average, ∼31000 high‐consensus, full‐length isoforms were obtained per fraction. By merging the fraction‐specific assemblies, a total of 72118 and 80735 nonredundant isoforms were identified in the cytoplasmic and nuclear fractions, respectively. However, less than 50% of the isoforms were detected in both fractions, indicating variable RNA populations in the two compartments^[^ ^61^ ^]^ (Figure S7, Supporting Information). We subsequently inspected the isoform heterogeneity by categorizing the isoforms in fraction‐specific assembly based on their splicing structure similarity to the transcripts from GENCODE V38 using SQANTI3^[^ ^37^ ^]^ (**Figure** **4**A, Figure S8, Supporting Information). The results showed that a large proportion (54–63%) of the isoforms matched with the reference annotation (isoforms classified as full splicing match). This high degree of overlap confirmed the assembly accuracy of LAFITE. We also found that the nucleus harbored more isoforms classified as NIC and ISM isoforms (novel isoforms with a combination of known splicing sites and novel isoforms matching the sequential section of reference isoforms, respectively; Figure 4A), implying more transcript forms derived from the same gene in the nucleus than in the cytoplasm. Together with the observation that genes in the nuclear fraction had more detectable isoforms (Figure S9, Supporting Information), these findings suggest an increased complexity of the nuclear transcriptome compared with the cytoplasmic transcriptome.

Previous studies have reported a low confidence in ISMs with a certain number of truncated isoforms.^[^ ^62^ ^]^ However, an in‐depth examination using orthogonal datasets evidenced that the ISMs detected by LAFITE had a significant percentage of overlap with CAGE peaks and poly(A) clusters (Figure S10, Supporting Information). By comparing the ISMs with the corresponding parent transcripts, we found that a substantial number of ISMs were classified as 3′ truncations that arose as a result of early polyadenylation events (Figure 4B). This suggested the critical omission of transcript variants with early polyadenylation signals in current gene annotation. For example, polyadenylation occurred after the third exon of one annotated transcript from the *MKKS* gene (*MKKS‐201*), leading to the formation of a novel transcript variant (Figure 4C). Subsequent 3′ rapid amplification of cDNA ends (RACE) and Sanger sequencing also confirmed the existence of this novel isoform (Figure S11, File S1, Supporting Information). In addition, we noted that more nuclear ISMs were characterized as 5′ truncations compared to the ISMs detected in the cytoplasm (Figure 4B). Further investigation of the position of the ISMs’ starting exon relative to the corresponding parent reference transcripts revealed that nuclear ISMs preferentially start in the central region of the gene (Figure 4D), indicating an increased incidence of alternative promoter usage in the nuclear transcriptome compared with the cytoplasmic transcriptome. For example, two ISMs from the *SLC2A4RG* gene that differed in their starting exons showed distinct subcellular enrichment patterns (Figure 4E). Collectively, this difference may be explained by the fact that alternative promoter usage may contribute to isoform diversity.^[^ ^63^ ^]^

Next, we examined and compared multiple genomic features between the cytoplasmic and nuclear fractions. The overall lengths of the transcripts from the nucleus were found to be greater than those from the cytoplasm for all four cell lines. However, there was no observable difference in the length distribution of exons and introns between the two compartments (Figure 4F, Figure S12, Supporting Information). Further investigation of the exon number revealed that isoforms with five or more exons were more specific to the nuclear fraction than the cytoplasmic fraction (Figure S13, Supporting Information). Therefore, this excessive exon use may be the fundamental cause of the predisposition of the nucleus for longer isoforms.

Additionally, we noticed generally longer 5′‐ and 3′‐UTRs in nuclear isoforms than in cytoplasmic isoforms. This is plausible as UTRs have been reported to be essential regulators of RNA stability. Long UTRs may protect RNA from degradation and hence promote nuclear export.^[^ ^64^ ^]^ However, the overall difference in 5′‐UTR length distribution almost disappeared when restricting the comparison to the isoforms identified in both fractions. By contrast, we still observed a slight shift in the 3′‐UTR length distribution, suggesting more alternative polyadenylation sites for transcripts in the nuclear fraction, leading to longer 3′‐UTRs (Figure 4F). For example, one isoform of the *TMED10* gene (*TMED10‐201*) showed distinct subcellular 3′‐UTR usage, with the longer 3′‐UTR only detected in the nuclear fraction (Figure 4G). A recent study confirmed that *miR‐7* specifically targets this longer 3′‐UTR and forms a sponge system, with the involvement of the circular RNA *CDR1as* to regulate cell proliferation.^[^ ^65^ ^]^

A previous study described a positive correlation between 3′‐UTR length and poly(A) tail length.^[^ ^66^ ^]^ Taking advantage of the full‐length RNA molecules captured by DRS, we performed poly(A) tail length estimation on our data using nanopolish,^[^ ^30^ ^]^ providing a subcellular‐resolved poly(A) profile. We then analyzed poly(A) tail length and 3′‐UTR length across all subcellular DRS datasets and found a similar weak correlation (Figure S14, Supporting Information). Nevertheless, the overall poly(A) tail length was consistently greater in nuclear fractions than in cytoplasmic fractions (*p* < 0.001, Mann–Whitney U test; Figure 4H). Similar patterns were observed when only considering the reads from isoforms assembled in both fractions (Figure 4I). This observation agrees with the previous finding that poly(A) length is functionally relevant to RNA exportation and stability.^[^ ^67^ ^]^ Taken together, our findings revealed inherent differences between cytoplasmic and nuclear fractions that required further investigation.

### 2.6 Subcellular DRS Reveals a Role of RNA Modification in Determining Subcellular Fate

**Figure 5:**
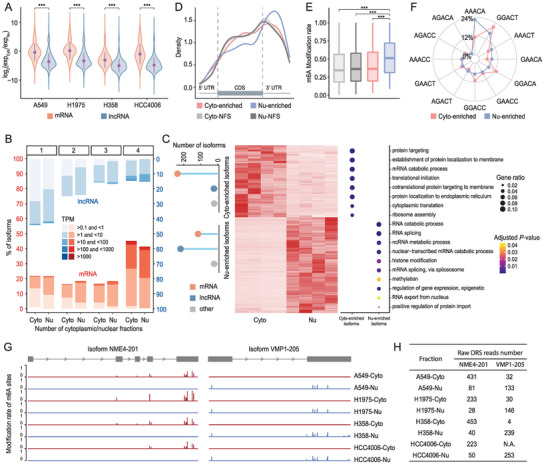
Integrative analysis of the subcellular transcriptome and epitranscriptome. A) Comparison of the isoform fraction distributions (isoform expression TPM ≥ 0.1 in both cytoplasm and nucleus) in different categories from four lung cancer cell lines (***p < 0.001, Mann‐Whitney U test). B) Isoform expression levels (in TPM) and the number of cytoplasmic or nuclear fractions in which the isoforms are expressed. The numbers at the top of the figure panel represent the number of cytoplasmic and nuclear fractions. C) Heatmap showing the diverse expression patterns of the isoforms enriched in the cytoplasmic and nuclear fractions. Plots on the left show the classification of cytoplasm‐enriched (Cyto‐enriched) and nucleus‐enriched (Nu‐enriched) isoforms. The dot plot on the right illustrates the Gene Ontology (GO) term annotation of the fraction‐enriched isoforms. D) Relative distribution of the m6A loci identified along the segments (5′‐UTR, coding sequence [CDS], and 3′‐UTR) of Cyto‐enriched, Nu‐enriched, and nonfraction‐specific (NFS) isoforms (Cyto‐NFS, Nu‐NFS). (E) Modification rate distribution of all m6A sites identified in the isoforms from different categories in D (***p < 0.001, Mann‐Whitney U test). F) Proportion of the conserved DRACH motifs identified from Cyto‐enriched, Nu‐enriched isoforms. G) Examples of two fraction‐enriched isoforms with distinct m6A modification patterns in the cytoplasmic and nuclear fractions. H) Number of full‐length DRS reads in each fraction that support the structure of the example isoforms in G.

To accurately evaluate the subcellular distribution of different RNA populations, we profiled and compared the relative expression levels of each transcript (TPM) in both cytoplasmic and nuclear fractions using paired Illumina short‐read data. We adopted a similar metric used in our previous study to define the prevalence of asymmetric transcript distribution.^[^ ^21^ ^]^ Isoforms with positive log_2_(TPM_cyto_/TPM_nu_) values were categorized as cytoplasmic, while those with negative values were categorized as nuclear. The results were consistent with our previous finding showing that mRNAs are not asymmetrically distributed between cytoplasmic and nuclear fractions, while lncRNAs show a significant nuclear predominance (*p* < 0.001, Mann–Whitney U test; **Figure** **5**A).^[^ ^21^ ^]^ In addition, the expression levels of mRNAs and lncRNAs across all cytoplasmic and nuclear fractions showed a complementary U‐shaped distribution pattern. Specifically, mRNAs tended to be ubiquitously expressed in all samples, whereas lncRNAs showed sample‐specific expression patterns (Figure 5B). We subsequently defined a group of fraction‐enriched isoforms with at least a two‐fold expression bias toward a specific fraction across all four cell lines, as quantified using both DRS and Illumina RNA‐seq data. Overall, 248 isoforms showed cytoplasmic enrichment, of which 95% were mRNAs. In contrast, lncRNAs accounted for more than 60% of the 330 nucleus‐enriched isoforms (Figure 5C). Gene Ontology (GO) analysis revealed that isoforms enriched in the cytoplasm were mainly involved in RNA translation and protein localization processes, while nucleus‐enriched isoforms were primarily related to RNA splicing and epigenetic regulation (Figure 5C). This, in combination with the asymmetric transcript distribution, indicated that the general intrinsic RNA subcellular localization did not vary by cell type. Hence, further investigation was performed to determine the underlying mechanism that determines the subcellular fate of RNA molecules.

Accumulating evidence has indicated that RNA modification plays an important role in regulation of the transcript subcellular localization.^[^ ^34^, ^68^ ^]^ Taking advantage of Nanopore DRS to preserve nucleotide modifications, we performed isoform‐level m6A modification identification in each fraction using nanom6A^[^ ^69^ ^]^ based on the pre‐distributed DRS reads from LAFITE. In total, 32742 and 31859 unique m6A modification sites located in 4837 and 4358 isoforms were detected in cytoplasmic and nuclear fractions, respectively (Table S4, Supporting Information). Consistent with previous observations, we found remarkable enrichment of m6A sites near the stop codons of cytoplasm‐enriched and nonfraction‐specific isoforms,^[^ ^70^ ^]^ whereas m6A sites in nucleus‐enriched isoforms were significantly more frequent in the middle of the 3′‐UTR (Figure 5D). Additionally, by quantifying the modification rate at each modified base, we observed a high m6A modification rate at sites in nucleus‐enriched isoforms, with a median value of 0.5 (Figure 5E). Further examination of the sequence features of the identified m6A sites also revealed a diverse usage of multiple DRACH motifs in different subcellular fractions (Figure 5F). These collective observations indicated that isoforms enriched in the nucleus may exhibit a distinctive m6A modification pattern. Indeed, we discovered several fraction‐enriched isoforms that exhibited a consistent fluctuating m6A modification pattern between cytoplasmic and nuclear fractions across all four cell lines. For example, isoforms of the *NME4* and *VMP1* genes, which had considerable full‐length DRS read counts in each fraction, only had detectable m6A modifications in the cytoplasmic fraction and nuclear fraction, respectively (Figure 5G,H). Altogether, these findings indicated a significant fractional difference in m6A modification, which may determine the subcellular localization of the RNA molecules.

### 2.7 Characterization of Alternative Splicing Events in Cytoplasmic and Nuclear Fractions

**Figure 6:**
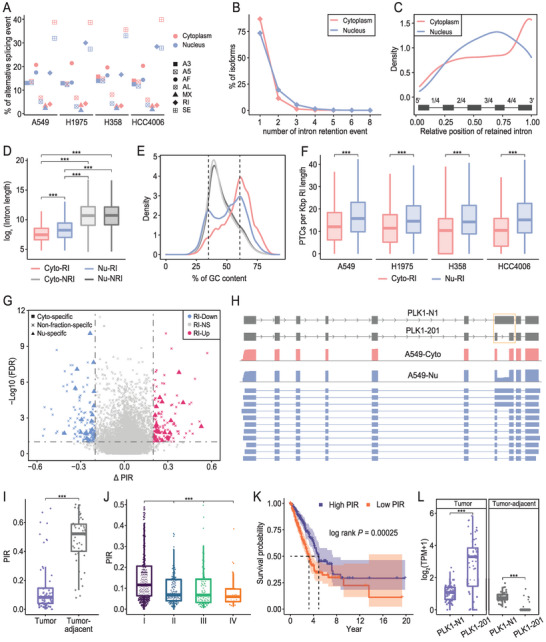
Characterization of alternative splicing in subcellular fractions. A) Number of detected alternative splicing (AS) events for the seven major classes shown in Figure S14A. B) Line plot showing the distribution of number of the retained introns (RIs) per isoform in merged cytoplasmic and merged nuclear assemblies. C) Relative positions of RIs in relation to the other introns in the transcript structure, calculated by dividing the intron position by the total number of introns in the transcript. D) Length distribution of the RIs detected in the cytoplasmic (Cyto‐RI) and nuclear (Nu‐RI) fractions and the nonretained introns (Cyto‐NRI, Nu‐NRI) (***p < 0.001, Mann‐Whitney U test). E) GC contents of the introns from different categories in D. F) Boxplots showing the number of in‐frame premature termination codons (PTCs) per Kbp intron length in Cyto‐RIs and Nu‐RIs (***p < 0.001, Mann‐Whitney U test). G) Volcano plot visualizing the differentially expressed RI events between tumor and tumor‐adjacent tissue datasets from the TCGA‐LUAD cohort, with symbols representing the fraction bias features of the RI events. RIs with |ΔPIR| > 0.2 and false discovery rate < 0.1 (Mann‐Whitney U test, Benjamini‐Hochberg correction) were considered as differentially expressed. Up‐ and down‐regulated RI events are depicted with red and blue symbols, respectively. RI events with no significant expression change are represented by grey symbols. H) Example of a novel RI events in *PLK1* gene (indicated in yellow box) specifically detected in the nuclear fraction. I) Boxplot showing the percent intron retention (PIR) values of the seventh intron of the *PLK1* gene in paired tumor and tumor‐adjacent tissues from the TCGA‐LUAD cohort (***p < 0.001, Mann‐Whitney U test). J) Boxplot showing the PIR values of the seventh intron of the *PLK1* gene across different stages of LUAD samples (***p < 0.001, Mann‐Whitney U test). K) Kaplan–Meier curves of 503 LUAD patients in TCGA‐LUAD cohorts with high and low PIR values for the seventh intron of the *PLK1* gene (*p* = 0.00025, Log‐rank test). L) Boxplot showing the expression levels of *PLK1‐N1* and the known isoform of *PLK1* (*PLK1‐201)* in tumor and tumor‐adjacent tissues from the TCGA‐LUAD cohort. TPM = transcripts per million (***p < 0.001, Mann‐Whitney U test).

To further characterize the subcellular transcriptome, we performed a comprehensive annotation of AS in cytoplasmic and nuclear fractions using the fraction‐specific transcriptome annotations generated by LAFITE. AS of precursor mRNA is prevalent in the eukaryotic transcriptome, leading to a diverse range of RNA and protein isoforms.^[^ ^71^ ^]^ There is increasing evidence showing that RNA isoforms produced by AS may have different regulatory functions and cellular localization. Using the SUPPA2 tool,^[^ ^72^ ^]^ thousands of AS events for each of the seven AS classes, alternative 3′‐acceptor (A3), alternative 5′‐donor (A5), alternative first exon (AF), alternative last exon (AL), mutually exclusive exon (MX), retained intron (RI), skipped exon (SE), were identified in each DRS dataset (Figure S15A, Table S5, Supporting Information). In line with the results of a previous study, SE stands out as the most frequent AS event across all fractions^[^ ^73^ ^]^ (**Figure** **6**A). In addition, SE events were prone to be detected in novel isoforms (Figure S15B, Supporting Information), suggesting an evident overlook of SE in current assembly emerged from short‐read sequencing data. Indeed, less than 20% of novel isoforms with SE events were identified in our Illumina RNA‐seq dataset (Figure S15C, Supporting Information). In contrast, we noticed a significant nuclear enrichment of RI events (Figure 6A, Figure S15B, Supporting Information). This remarkable fluctuation provides further support for the critical role of RI events in regulating the RNA subcellular fate.^[^ ^22^ ^]^ Given the marked difference in the number of RI events between the two fractions, the following analyses focused on RI events.

We started by investigating multiple characteristics of RIs, including their frequency, position, length, and GC content. We found a relatively higher complexity of RI events in nuclear fractions compared with cytoplasmic fractions, as indicated by a greater proportion of isoforms with two or more RI events (26.5% in the nucleus vs 13.1% in the cytoplasm; Figure 6B, Figure S16A, Supporting Information). This reinforces our aforementioned findings that the nucleus has a more complex transcriptome, in which RI events appear to be key contributors to transcriptome diversity.^[^ ^74^ ^]^ Further characterization of the genomic distribution of RIs revealed distinct relative positions of the RIs in transcripts from the cytoplasmic and nuclear fractions. Specifically, we observed a strong 3′ end bias for RIs in the cytoplasmic fraction, as previously described,^[^ ^75^ ^]^ whereas RIs in the nuclear fraction were more likely to be in the center of the isoform body (Figure 6C, Figure S16B, Supporting Information). Previous research has shown that RIs are significantly shorter and have a higher GC content than nonretained introns.^[^ ^76^ ^]^ We confirmed these observations; however, RIs from the nuclear fraction were still longer than those from the cytoplasmic fraction and had a second peak of GC content approaching the GC content of nonretained introns (Figure 6D,E, Figure S16C,D, Supporting Information). Taken together, these results indicated a broad divergence in RIs between cytoplasmic and nuclear fractions, as well as the possibility that some introns preserved in nuclear isoforms have unexpected features.

It has been reported that many introns are included in polyadenylated RNA but undergo post‐transcriptional splicing before entering the cytoplasm.^[^ ^77^ ^]^ This may occur because the polyadenylation of these RNAs is still in progress, and splicing is completed once polyadenylation has been accomplished. Therefore, these RNAs are expected to have long poly(A) tails due to the extended polyadenylation process. We confirmed this trend in nuclear isoforms with RIs, which also showed a broader poly(A) tail length distribution than the cytoplasmic isoforms with RIs and the remaining isoforms without RIs (*p* < 0.001, Mann–Whitney U test; Figure S17A, Supporting Information). This implies that the corresponding RNA molecules in the nuclear fraction may undergo polyadenylation to a different extent. These findings may reflect the false annotation of RIs due to unspliced introns in nuclear DRS reads, leading to differences in RIs between the two fractions. Indeed, novel RIs in the nucleus showed significantly lower relative expression levels (percent intron retention [PIR]) than known RIs (*p* < 0.001, Mann–Whitney U test). However, this was not observed for cytoplasmic RIs (Figure S17B, Supporting Information). This further suggests a lower level of confidence in the annotation of novel RIs in nuclear fractions. Nevertheless, we still observed a similar striking divergence in RIs between cytoplasmic and nuclear fractions when restricting the analyses to RIs with a higher read coverage (≥5, Figure S18, Supporting Information), indicating a functional dependency of RIs in the nuclear fraction.

RIs are known to contain in‐frame premature termination codons (PTCs), leading to the nonsense‐mediated mRNA decay (NMD) of RI‐containing transcripts. This surveillance mechanism has been revealed as a major controller of gene expression under normal conditions.^[^ ^78^ ^]^ By comparing the density of PTCs in cytoplasmic RIs versus nuclear RIs, we found that nuclear RIs possessed a significantly higher PTC density across all four cell lines (Figure 6F). This, in combination with the above findings, implied that these introns in the nuclear fraction may be retained to fulfill important functions, including regulation of gene expression.

### 2.8 Fraction‐Enriched Retained Introns Are Correlated with Tumor Progression

To investigate the potential biological effect of the identified RI events on cancer progression, we extended our analysis by profiling their expression levels (PIR) in all 596 RNA‐seq datasets from the TCGA‐LUAD cohort. A comparison of paired primary tumor and tumor‐adjacent tissue samples identified 255 differentially expressed RIs (DERIs; 146 novel and 109 known) from 225 genes with the criteria of |ΔPIR| > 0.2 and a false discovery rate < 0.1 (Mann–Whitney U test, Benjamini‐Hochberg correction; Figure 6G, File S2, Supporting Information). Hierarchical clustering of all 596 datasets based on the expression profile of the DERIs revealed a clear separation of the tumor and nontumor groups, reflecting a reliable detection of the dysregulated RIs (Figure S19, Supporting Information). Notably, 40 DERIs were found to be specifically detected in the nuclear fraction, and many of these were from known cancer‐related genes, including *AGRN*, *DKC1*, *PKMYT1*, *PLK1*, and *MARS1*. In contrast, only one DERI was assigned as cytoplasm‐specific, suggesting an active role of nuclear AS in cancer progression (Figure 6G).

We noticed a novel RI event, the retention of the seventh intron of *PLK1* gene, which was specifically detected in a novel isoform (*PLK1‐N1*) across all nuclear fractions and showed a significantly decreased inclusion level (PIR) in the tumor group (Figure 6H,I). *PLK1* is broadly involved in cancer development and is a potential target for clinical therapy.^[^ ^79^ ^]^ We further analyzed the patients’ pathological stage in relation to the PIR value and found a negative correlation between PIR value and tumorigenicity (Figure 6J). Additionally, the results of Kaplan–Meier survival analysis also indicated that patients with a low PIR value exhibited an unfavorable prognosis (Figure 6K). These findings implied that changes in the inclusion of the seventh intron of *PLK1* may have a large effect on tumorigenesis. Given the complete form of the alternatively spliced isoforms revealed by DRS, we found an in‐frame PTC located on this RI that may drive *PLK1‐N1* to NMD and control the expression of *PLK1* gene (Figure S20, Supporting Information). Therefore, the downregulation of this RI might be a key factor leading to the aberrant expression of the dominant *PLK1* isoform (*PLK1‐201*) by splicing *PLK1‐N1* to *PLK1‐201* (Figure 6H), thereby affecting tumor progression. Indeed, we observed opposite expression patterns of *PLK1‐N1* and *PLK1‐201* in tumor and tumor‐adjacent tissues, which provided further support to our hypothesis (Figure 6L).

Overall, using a combination of fractionation DRS data and RNA‐seq data from tumor samples, we revealed the functional importance of fraction‐enriched RIs which could serve as an additional explanation for the involvement of genes in cancer development. In addition, the direct linkage between AS and specific isoforms revealed by Nanopore DRS provides an alternative approach to illustrate the detailed mechanism of how AS contributes to disease progression.

## 3 Discussion

A broad landscape of subcellular distribution of RNA is a pre‐requisite for understanding their regulatory function in cellular processes. Previous subcellular transcriptome studies using FISH or short‐read sequencing have revealed informative RNA compartmentalization;^[^ ^13^, ^15^ ^]^ nonetheless, none of these studies have achieved resolution at the full‐length transcriptome‐wide level due to the major challenges with throughput and read length. Here, we conducted a fractionation followed by DRS using four model cell lines (A549, H1975, H358, and HCC4006) to capture the full‐length cytoplasmic and nuclear transcriptomes. However, the throughput limitations of DRS libraries still challenged the use of currently available long‐read transcriptome assembly tools, particularly for low‐abundance transcripts. Therefore, we developed LAFITE, a dedicated pipeline to define high‐consensus, full‐length isoforms despite low coverage.

In contrast to short‐read RNA‐seq, in which transcripts can only be inferred from read overlap, the principle of long‐read transcriptome assembly should be to truly restore the transcripts included in the library. Thus, the key challenge for transcript annotation from long‐read data is to correct the artifacts and remove truncated reads resulting from a high error rate and read fragmentation.^[^ ^45^ ^]^ Current state‐of‐the‐art tools, including FLAIR, StringTie and TrackCluster, rely excessively on sequence coverage to filter these sequencing noises. However, this simple operation cannot effectively remove these false positives, especially for low‐quality datasets with pervasively truncated reads. In addition, a high read coverage requirement substantially limits the downstream analyses to those highly expressed transcripts. In contrast, LAFITE achieved better utilization of information recorded in the sequence data and reference annotation, and employed an integration‐and‐filtering strategy by considering the presence of the polyadenylation event of individual read, the reliability of each splicing junction, and the confidence levels of the read start site and read end site to comprehensively assess isoform fidelity, thereby minimize the dependence on sequencing depth. Consequently, LAFITE exhibits marked improvements in isoform detection and low‐abundance transcript rescue compared to other commonly used tools, as evidenced by analyses of DRS and other orthogonal datasets. In combination, LAFITE enables an unprecedented opportunity to identify full‐length isoforms, including low‐abundance isoforms that have not previously been annotated.

By applying LAFITE to our subcellular DRS data, we identified 72118 and 80735 nonredundant isoforms in the cytoplasmic and nuclear fractions, respectively. Surprisingly, less than 50% of these nonredundant isoforms matched the reference gene annotation, indicating a high level of transcriptome diversity revealed using the combination of LAFITE and DRS. Notably, the coverage statistics revealed that more than 70% of novel isoforms detected by LAFITE had fewer than two full‐length reads, implying the major overlook of low‐abundance isoforms in current annotations. Subsequent molecular assays further uncovered the oncogenic function of one novel low‐abundance isoform from the *AKT1* gene (*AKT1‐N1*) that contrasted with the known function of *AKT1*,^[^ ^56^ ^–^ ^58^ ^]^ indicating a double‐agent attribute of *AKT1* with both oncogenic and tumor‐suppressor functions. Given that tissue‐specific gene expression and splicing patterns are ubiquitous,^[^ ^80^, ^81^ ^]^ a comprehensive characterization of the isoform output of individual genes may change our understanding of their biological functions.

Previous studies have reported that the nuclear transcriptome is more complex than the cytoplasmic transcriptome.^[^ ^32^ ^]^ Taking advantage of the ability of Nanopore DRS to profile all transcript elements, we refined this conclusion to the isoform level, as we observed more extensive isoform diversity and longer poly(A) tail and UTR in the nuclear fraction. An astonishing observation arising from our study was the fraction‐biased 3′‐UTR length of isoforms detected in both compartments. This alternative 3′‐UTR usage may provide an additional mechanism for fraction‐specific post‐transcriptional regulation, as a longer 3′‐UTR facilitates the binding of RNA‐binding proteins and miRNAs.^[^ ^82^ ^]^ For example, the dominant isoform of *TMED10 (TMED10‐201*) had considerable read counts in the two fractions but possessed a longer 3′‐UTR specifically in the nucleus, and this longer 3′‐UTR has been associated with cell proliferation by acting as an *miR‐7* sponge.^[^ ^65^ ^]^ Although the functional significance of the inconsistent 3′‐UTR lengths in the two fractions remains to be determined, these widespread phenomena highlight the value and importance of investigating subcellular variation at the isoform level.

In addition to sequence‐level differences, we also revealed a divergence in m6A modifications between the two fractions according to the base modification information documented in the DRS dataset. Unlike previous studies that limited the characterization of m6A modifications to the gene level, we significantly broadened the complexity of the methylome by mapping isoform‐level m6A sites on a transcriptome‐wide scale. Accordingly, we report a distinct m6A modification pattern for nucleus‐enriched isoforms, in terms of their relative position, the degree of modification, and their DRACH motif usage. These distinct characteristics confirmed the critical role of the m6A modification in affecting the subcellular fate of RNA molecules. Collectively, the combination of transcript complexity revealed by LAFITE and isoform‐level m6A identification has provided new avenues for the study of subcellular transcriptomes. With the development of approaches for decoding other modification types from DRS data, this integration strategy has the potential to elucidate the hidden interactions between the transcriptome and the epitranscriptome.

Another important discovery in this study is the genuine differences in the position, length, and GC content of RIs between the cytoplasmic and nuclear fractions. The exact mechanism accounting for these differences has yet to be elucidated. However, a possible explanation is that the RI‐mediated control of gene expression requires the retention of unexpected introns within the nucleus, as evidenced by:^[^ ^1^ ^]^ nuclear RIs showing a higher in‐frame PTC density that would promote the degradation of the parent isoforms via NMD;^[^ ^2^ ^]^ more isoforms in the nucleus harboring multiple RI events (26.5% in the nucleus vs 13.1% in the cytoplasm), which significantly increases the possibility of NMD; and^[^ ^3^ ^]^ RIs in the nucleus tending to be more common in the central region of the isoforms, leading to the presence of longer 3′‐UTRs and subsequent miRNA‐induced translation repression. Such an error‐correction strategy has been demonstrated to be an efficient procedure to post‐transcriptionally safeguard gene expression.^[^ ^75^ ^]^ Furthermore, using RNA‐seq data from the TCGA‐LUAD cohort, we found 255 RIs that were differentially expressed between tumor and tumor‐adjacent tissue, of which 40 and 1 showed a strong nuclear and cytoplasmic expression bias, respectively. This sharp contrast further reflects the functional importance of nuclear RIs and may help elucidate the underlying mechanisms employed by genes involved in disease progression.

In conclusion, we demonstrated that nanopore DRS is well suited for studying subcellular transcriptomes by linking different transcript elements to the isoform level, thereby allowing the systematic investigation of the biological significances related to the subcellular distribution of RNA molecules. Moreover, the newly developed pipeline, LAFITE, will serve as a valuable resource for long‐read transcriptome analysis.

## 4 Experimental Section

### Cell Culture

The human lung cancer cell lines A549 (American Type Culture Collection [ATCC], Manassas, VA, USA; CCL‐185), H1975 (ATCC, CRL‐5908), H358 (ATCC, CRL‐5807), and HCC4006 (ATCC, CRL‐2871) were cultured in complete RPMI‐1640 medium (Gibco, Waltham, MA, USA; Cat‐11875093) supplemented with 10% fetal bovine serum (Gibco, Cat‐10270106) and 1% penicillin‐streptomycin (Gibco, Cat‐15070063) and maintained at 37 °C in a humidified incubator supplemented with 5% CO_2_. All cell lines were tested and found to be free of mycoplasma contamination prior to being used in the experiments.

### Subcellular Fractionation and Total RNA Isolation

Subcellular fractionation was performed according to the method described in our previous study.^[^ ^21^ ^]^ Briefly, a cell pellet of approximately 10^8^ cells was resuspended in 4 mL of hypotonic lysis buffer (RLN buffer [Qiagen, Hilden, Germany] supplemented with 0.5% NP‐40) and incubated on ice for 5 min. The lysate was then centrifuged at 1000 × g for 4 min at 4 °C to separate the nuclear (pellet) and cytoplasmic (supernatant) fractions. The cytoplasmic fraction was removed and centrifuged at 11000 × g for 2 min at 4 °C to remove residual nuclei. The pellet containing the nuclei was rinsed twice with 4 mL of hypotonic lysis buffer to generate the purified nuclear fraction. Finally, each fraction was subjected to total RNA extraction using a TRIzol/RNeasy hybrid protocol, as previously described.^[^ ^83^ ^]^

### qRT‐PCR Validation of Fraction‐Specific RNA Extraction

To confirm successful fractionation, qRT‐PCR was performed to measure the relative expression of marker genes in both fractions. Specifically, 1 µg of total RNA from paired cytoplasmic and nuclear fractions was treated with DNase I (Invitrogen, Carlsbad, CA, USA) to eliminate contaminating gDNA, followed by cDNA synthesis using PrimeScript RT Master Mix (Takara, Tokyo, Japan) in a total volume of 20 µL. The cDNA products were then subjected to qPCR using TB Green Premix Ex Taq II (Takara) and a CFX9 Real‐Time PCR Detection System (Bio‐Rad, Hercules, CA, USA) according to the manufacturer's instructions. All qRT‐PCR mixture volumes were 10 µL and contained 1 µL of cDNA and 0.2 × 10^−6^ m of each primer. The PCR primer sequences are listed in File S3 (Supporting Information).

### Nanopore DRS Library Preparation and Sequencing

Total RNA samples (200 µg) were enriched for poly(A)+ RNA using a Poly(A)Purist MAG Kit (Thermo Fisher Scientific, Waltham, MA, USA) following the manufacturer's instructions. The quality and quantity of the samples were assessed using a NanoDrop 1000 spectrophotometer (Thermo Fisher Scientific) and a Qubit 4 Fluorometer (Thermo Fisher Scientific), respectively. One thousand nanograms of poly(A)+ RNA were aliquoted for library preparation using a Nanopore DRS kit (Oxford Nanopore Technologies, Oxford, UK; SQK‐RNA002) according to a standard protocol. Libraries were loaded onto R9.4.1 SpotON Flow Cells and sequenced using a MinION system (Oxford Nanopore Technologies) for 72 h.

### Illumina RNA Sequencing

Strand‐specific total rRNA‐depleted RNA‐seq libraries were constructed and sequenced using a 150 bp paired‐end strategy on a NovaSeq 6000 platform (Illumina, San Diego, CA, USA) by Novogene Technology Co. (Beijing, China). An average of 60 million pair‐end reads were generated from eight fractions.

### Sequence Data Processing—Reference Genomes and Annotations

The primary assemblies of GRCh38 and GRCm39 and GENCODE V38 and GENCODE VM28 were used as the reference genomes and annotation references for human and mouse, respectively. The TAIR10 and WBcel235 assemblies and the full corresponding annotations (Ensembl 50, Ensembl 103) obtained from Ensembl were used as the reference genomes and annotation references for *Arabidopsis* and *C. elegans*, respectively. Additionally, the reference annotations for human (GCF_000001405.39), mouse (GCF_000002985.6), *Arabidopsis* (GCF_000001735.4), and *C. elegans* (GCF_000002985.6) were downloaded from RefSeq for LAFITE assessment.

### Sequence Data Processing—Nanopore DRS Data Processing

The raw sequencing signals for all samples initially underwent base‐calling and adapter trimming using Guppy (version 5.0.17).^[^ ^84^ ^]^ The resulting sequence was aligned to the corresponding reference genome sequence using minimap2 (version 2.17)^[^ ^85^ ^]^ with the guide of the reference annotation. The alignment files were then subjected to reference‐guided transcriptome assembly using FLAIR,^[^ ^38^ ^]^ LAFITE, StringTie (version 2.1.4),^[^ ^39^ ^]^ and TrackCluster.^[^ ^40^ ^]^ Specifically, all tools were run under the default parameters with the supplement of DRS alignment file and reference annotation. StringTie was used with the “‐L” parameter setting for long‐read datasets. LAFITE was also supplied with the read polyadenylation status estimated from raw signal by nanopolish.^[^ ^30^ ^]^

### Sequence Data Processing—Illumina RNA Sequencing Data Processing

All raw short‐read datasets were first trimmed to remove adapters and low‐quality bases using Cutadapt (version 3.1).^[^ ^86^ ^]^ The resulting clean reads were then aligned to the reference genome using STAR (version 2.7.3a),^[^ ^87^ ^]^ followed by *de novo* transcriptome assembly using StringTie (version 2.1.4),^[^ ^39^ ^]^ with the guide of the reference annotation. The details of the read statistics are provided in Table S6 (Supporting Information).

### Performance Evaluation Metrics

We adopted the same metrics used by previous studies to evaluate the performance of different assemblers on the DRS data.^[^ ^39^, ^46^ ^]^ The precision and recall were calculated based on the following formulas

(1) $$\mathrm{Precision}=\frac{\mathrm{TP}}{\mathrm{TP}+\mathrm{FP}}$$

(2) $$\mathrm{Recall}=\frac{\mathrm{TP}}{\mathrm{TP}+\mathrm{FN}}$$

where TP is the number of true positives (assembled transcripts matching with the reference), FN is the number of false negatives (reference transcripts absent from the assembly), and FP is the number of false positives (novel assembled transcripts in comparison with the reference annotation). For the assemblies generated from real transcriptome datasets, the FP should be referred to as “novel predictions” due to the incompleteness of the reference gene annotation. Nonetheless, these metrics have remained for the consistency of the study.

### Validation of TSS and TES Using Orthogonal Datasets

We used multiple orthogonal datasets to validate the TSSs and TESs of the transcripts, including 5′‐end CAGE/SAGE peaks and 3′‐end poly(A) cluster. The processed CAGE or SAGE peaks were obtained from FANTOM5 (human and mouse),^[^ ^88^ ^]^ Le et al. (*Arabidopsis*),^[^ ^89^ ^]^ Saito et al. (*C. elegans*)^[^ ^49^ ^]^ and used to validate the TSSs; and the poly(A) cluster were retrieved from PolyASite 2.0 (human, mouse, *C.elegans*)^[^ ^90^ ^]^ and PlantAPAdb (*Arabidopsis*)^[^ ^91^ ^]^ for TES validation. TSS and TES were defined as “supported by orthogonal datasets” if it overlapped with a +/‐ 25 bp CAGE/SAGE peak and poly(A) cluster, respectively.

### Alternative Splicing Event Identification and Quantification

AS events were identified using the *generateEvent* command in SUPPA2.^[^ ^72^ ^]^ An AS event was considered to be novel if it was not documented in GENCODE V38. The expression level (PIR) of RIs in each fraction was then quantified using the *psiPerEvent* command in SUPPA2, with the incorporation of the transcript expression profile generated by Nanocount.^[^ ^53^ ^]^ Similarly, we estimated the PIR value of each RI in TCGA‐LUAD datasets using the transcript expression matrix computed by kallisto (version 0.46.0).^[^ ^92^ ^]^

### Single‐Isoform‐Level m6A Modification Identification

To identify m6A modification sites at the single‐isoform level, we assigned full‐length DRS reads and raw signal data to each isoform based on the splicing structure. nanom6A^[^ ^69^ ^]^ was then used to identify the m6A modifications in each isoform. Isoforms with fewer than five DRS reads were excluded from the analysis to minimize the number of low‐confidence loci.

### GO Enrichment Analysis

To interpret the potential biological functions of fraction‐enriched isoforms, we performed GO term enrichment analysis based on the parent gene names using the R package clusterProfiler.^[^ ^93^ ^]^ *p*‐values were adjusted using the Benjamini–Hochberg method. An adjusted *p*‐value < 0.01 was used as the threshold for determining significant GO terms.

### Survival Analysis

To assess the prognostic value of the candidate isoform or RI, we explored the relationship between their expression and overall survival of patients in TCGA‐LUAD cohort. The expression level of each isoform in TCGA‐LUAD tumor tissues was quantified with the corresponding RNA‐seq datasets using kallisto.^[^ ^92^ ^]^ Patients with survival data were classified into high‐risk and low‐risk groups based on the expression level of the candidate isoform or RI. Kaplan–Meier survival analysis was then performed, and a log‐rank test was used to estimate the differences in overall survival times between patients in the two groups.

### Validation of Novel Isoforms

Six novel isoforms from the genes *AKT1*, *BCL7B, ERCC1*, *RAB10*, *TGFB1*, and *TSPAN15* were validated using PCR followed by gel electrophoresis. Briefly, 1 µg of total RNA from the cytoplasmic fraction of A549 cells was treated with DNase I (Invitrogen) to eliminate contaminating gDNA. cDNA synthesis was then performed in a total volume of 20 µL using the PrimeScript RT Master Mix (Takara). Two microliters of the cDNA product were used for PCR amplification using Platinum SuperFi PCR Master Mix (Invitrogen) with a primer concentration of 0.5 × 10^−6^ m and a final volume of 50 µL. All primer sequences are listed in File S3 (Supporting Information). The resulting PCR products were electrophoresed on a 2.0% agarose gel and purified using a PureLink Quick Gel Extraction Kit (Invitrogen) prior to Sanger sequencing.

### 3′ RACE PCR

To validate the early polyadenylation event occurring on *MKKS*, 3′ RACE touchdown PCR was used to specifically amplify the 3′ end of the novel isoform harboring this early polyadenylation event. For this purpose, 1 µg of total RNA from the cytoplasmic and nuclear fractions A549 cells was treated with DNase I (Invitrogen) and used for first‐strand cDNA synthesis using an oligo (dT)‐anchored universal primer and a PrimeScript RT‐PCR Kit (Takara). The resulting cDNA product was used for touchdown PCR with a gene‐specific forward primer and a universal reverse primer. To increase the reaction sensitivity and specificity for the target amplicon, a nested PCR was performed using the 3′ RACE product with a nested gene‐specific primer and a nested universal primer. Both 3′ RACE and nested PCR were performed using Platinum SuperFi PCR Master Mix (Invitrogen) in a 50 µL reaction volume with a primer concentration of 0.5 × 10^−6^ m. The PCR product from the nested PCR was separated on a 2.0% agarose gel and purified using a PureLink Quick Gel Extraction Kit (Invitrogen) prior to Sanger sequencing. All primer sequences are listed in File S3 (Supporting Information).

### Vector Construction

The full‐length sequence of *AKT1‐N1* was amplified using overlap extension PCR.^[^ ^94^ ^]^ Briefly, 1 µg of total RNA from A549 cells was treated with DNase I (Invitrogen), followed by cDNA synthesis using PrimeScript RT Master Mix (Takara). The cDNA products were subjected to PCR amplification with three different primer sets to generate three fragments of *AKT1‐N1* with overlapping sequences for overlap extension. The forward primer for fragment 1 and the reverse primer for fragment 3 were annealed with the restriction site sequences for downstream digestion. The primary PCR products were then gel‐purified using a PureLink Quick Gel Extraction Kit (Invitrogen), and the purified fragments were quantified using a Qubit 4 fluorometer (Thermo Fisher Scientific). Then, 10 ng of each type of fragment were combined for overlapping PCR to generate full‐length *AKT1‐N1* without additional primers. Subsequently, the forward primer for fragment 1 and the reverse primer for fragment 3 were added to the reaction for final PCR amplification. All PCR assays were performed using Platinum SuperFi PCR Master Mix (Invitrogen) in a volume of 50 µL with a primer concentration of 0.5 × 10^−6^ m. Finally, the amplicon and pcDNA3.1 Myc‐His A vector (Invitrogen) were digested with KpnI (New England Biolabs, Ipswich, MA, USA; #R3142) and XbaI (New England Biolabs, #R0145) restriction enzymes and ligated using T4 DNA ligase (New England Biolabs, #15224017). Following cloning, colony selection and Sanger sequencing were performed to confirm the vector construct. All primer sequences are listed in File S3 (Supporting Information).

### Wound‐Healing Assay

Cell migration and motility were investigated using a wound‐healing assay. Briefly, A549 cells were seeded in 24‐well culture plates (1.5 × 10^5^ cells per well) and incubated overnight. The cells were then transfected with 500 ng of the *AKT1‐N1* vector and the control vector using Lipofectamine‐3000 reagent according to the manufacturer's protocol (Thermo Fisher Scientific). After 24 h of incubation, the confluent cell monolayer was scratched using a pipette tip to create a wound at the midline of the culture well. The cells were then rinsed once with phosphate‐buffered saline to remove the detached or dead cells and then replenished with fresh RPMI‐1640 medium. Subsequently, cells were immediately imaged under an inverted microscope (Nikon, Tokyo, Japan; TE300) at different time points. Cell migration was calculated by the change in the size of the wound at different time points. Data were normalized to the control vector.

## Conflict of Interest

The authors declare no conflict of interest.

## Author Contributions

J.Z. performed the experiments, designed the methodology, analyzed the data, and wrote the manuscript. X.L. contributed to the experiments on fractionation sequencing. T.H.L. and A.C.K.L. contributed to the experiments on molecular assays. Y.C. contributed to the methodology development. E.Y.H.C. provided expertise and feedback. W.C.S.C. provided cell lines, expertise and feedback. T.F.C. supervised the study, acquired funding, and wrote and revised the manuscript. All authors read and approved the final manuscript.

## Supporting information

Supporting Information

Supporting Information

Supporting Information

Supporting Information

## Data Availability

The raw sequence files generated in this study have been uploaded to Sequence Read Archive under the accession ID: PRJNA843514. The DRS data for SIRV E0 mix, SIRV E2 mix, Mouse ESC and C. elegans males can be found on SRA with the accession: SRR6058584, SRR6058583, SRR11550261 and ERR3245476, respectively. The DRS data for Arabidopsis buds were acquired from Zhang et al.^[^ ^31^ ^]^ with the author authorization. The DRS data for GM12878 cell line was obtained from Workman et al.^[^ ^30^ ^]^ (https://github.com/nanopore‐wgs‐consortium/NA12878/blob/master/RNA.md). The short‐read RNA‐seq datasets corresponding to Arabidopsis buds, Mouse ESC, GM12878 cell line and C. elegans males was obtained from SRA with following accession: SRR10399319, SRR10399320, SRR10399321, SRR10399322 (Arabidopsis buds); SRR3290186, SRR3290187, SRR3290189, SRR3290191, SRR3290192, SRR3290194, SRR3290195, SRR3290197, SRR3290210 (Mouse ESC); SRR14637068, SRR14637069, SRR14638511, SRR14638512, SRR14638513, SRR14638514 (GM12878 cell line); SRR3657229, SRR3657230, SRR3657231, SRR3657232, SRR3657233 (C. elegans males). The source code of LAFITE is available at https://github.com/TF‐Chan‐Lab/LAFITE/.
