# Supplementary material for: LAFITE Reveals the Complexity of Transcript Isoforms in Subcellular Fractions
Source: Adv Sci (Weinh). 2022 Dec 3;10(3):2203480. doi: 10.1002/advs.202203480 (PMC9875686; doi:10.1002/advs.202203480)
Supplement: Supplementary file 1 — Supporting Information [file ADVS-10-2203480-s004.pdf]

LAFITE reveals the complexity of transcript isoforms in subcellular fractions

Jizhou Zhang, Xiao Lin, Yuelong Chen, Tsz-Ho Li, Alan Chun-Kit Lee, Eugene Yui-Ching Chow, William Chi-Shing Cho, Ting-Fung Chan*

*Corresponding author. Email: tf.chan@cuhk.edu.hk

This file includes:

Figure S1 to S20
Tables S1 to S6

Other Supporting Materials for this manuscript include the following:
Files S1 to S3

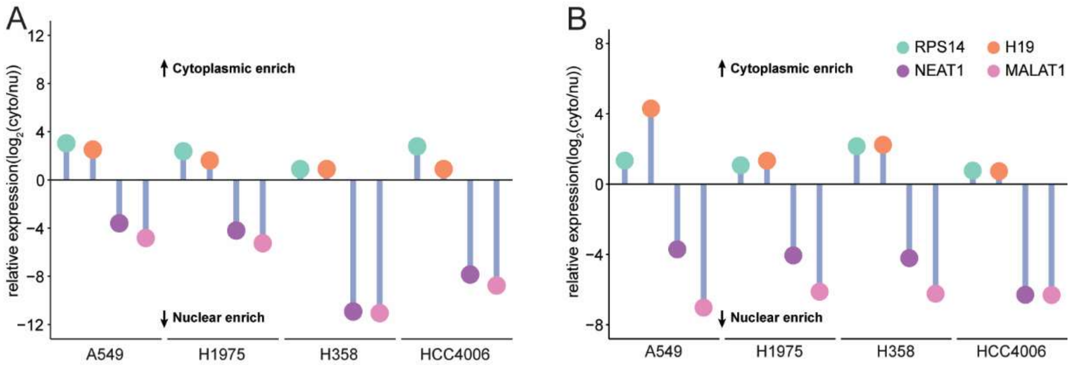

Figure S1. Relative cytoplasm-to-nucleus expression of cytoplasmic maker genes (*H19*, *RPLS14*) and nuclear marker genes (*MALAT1*, *NEAT1*) quantified by using Illumina RNA-seq data **(A)** and Nanopore DRS data **(B)**. Each fraction was sequenced one time with Illumina RNA-seq and Nanopore DRS, respectively.

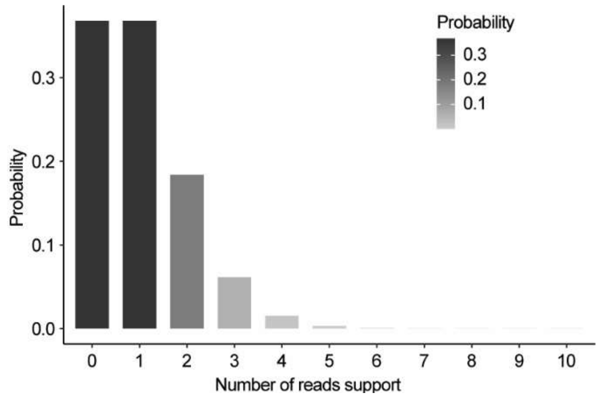

Figure S2. Simulation results revealed the probability distribution corresponding to the number of the captured DRS reads for a transcript with 1 TPM expression in a general Nanopore DRS run (1 M reads throughput).

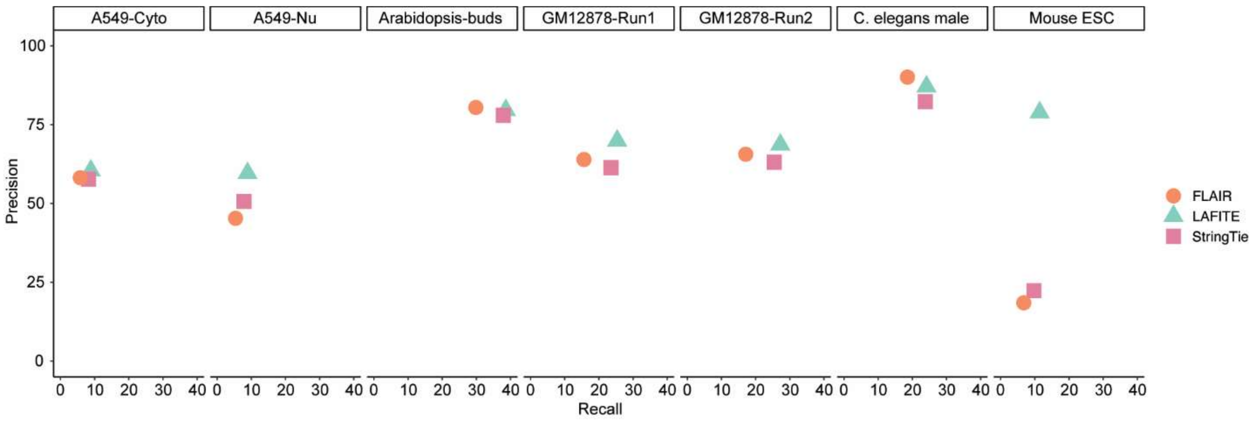

Figure S3. Precision and recall plot depicting the performance of the assemblers (FLAIR, LAFITE and StringTie) with seven datasets when using gene annotation from Ensembl

(*Arabidopsis* and *C. elegans*) or GENCODE (A549-Cyto, A549-Nu, GM12878-Run1 and GM12878-Run2).

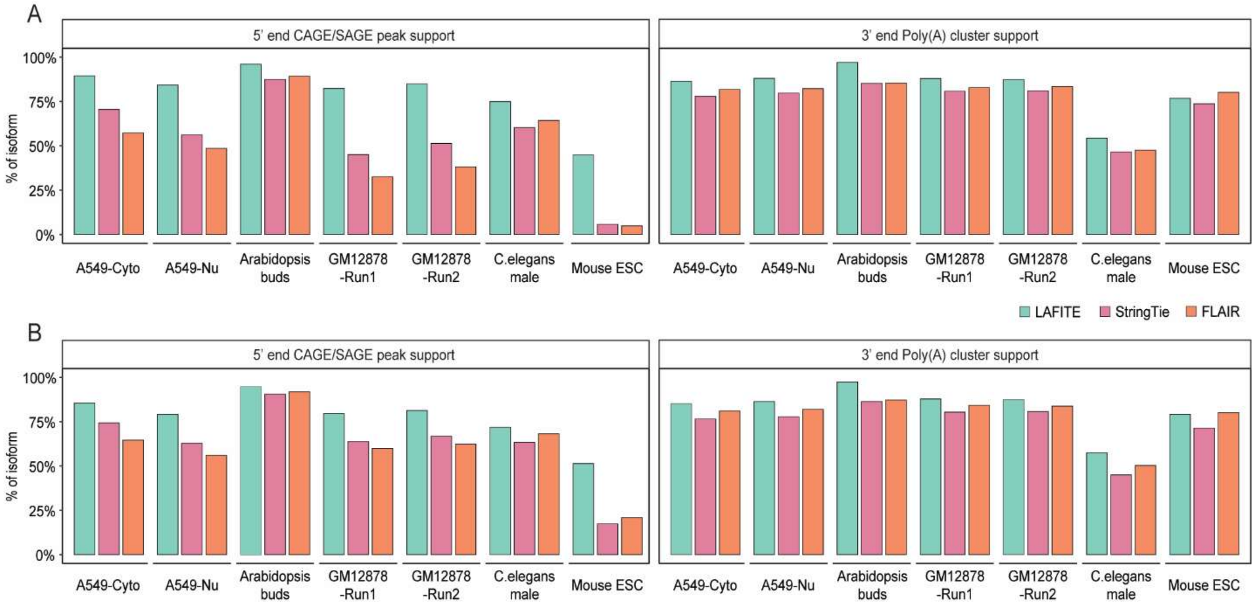

Figure S4. The proportion of the TSS and TES of the novel isoforms **(A)** and low-abundance isoforms (with two or fewer reads support) **(B)** in seven datasets supported by CAGE/SAGE peaks, and the presence of a poly(A) clusters, respectively.

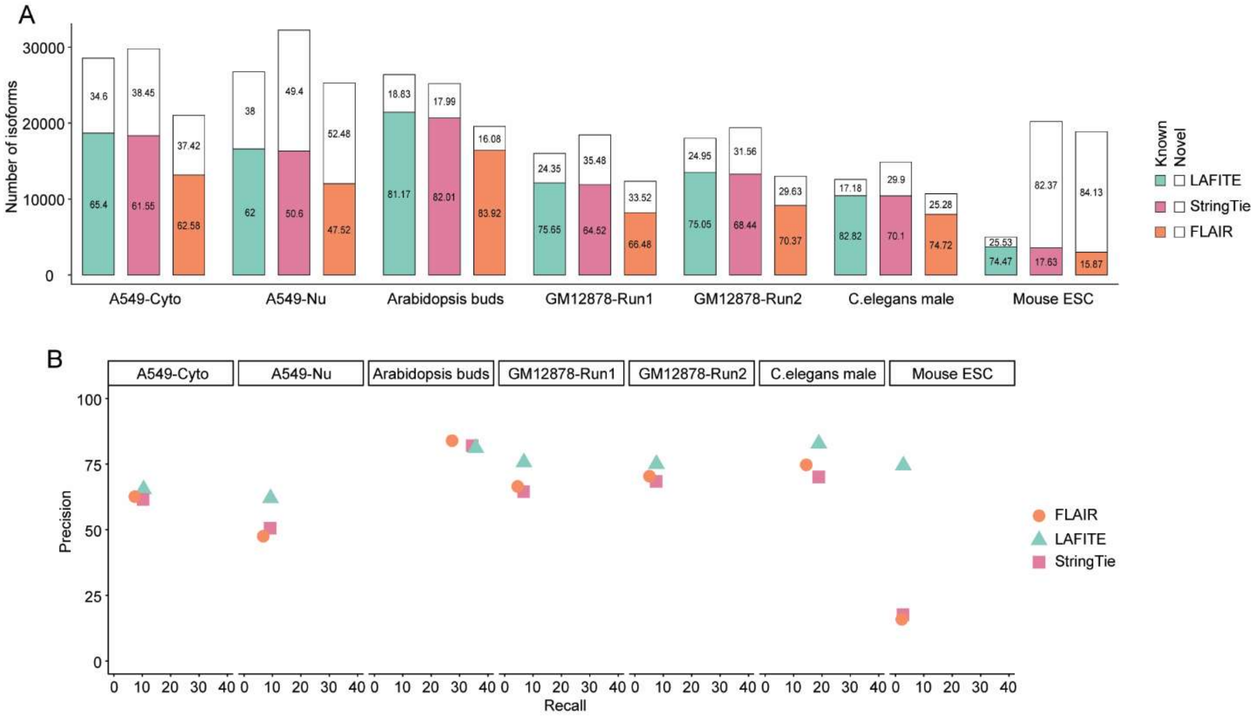

Figure S5. **Comparisons of performance of the assemblers (FLAIR, LAFITE and StringTie) on seven datasets under the guide of the RefSeq gene annotation.** **(A)** Stacked bar plot depicting the performance of LAFITE, FLAIR, and StringTie with seven DRS datasets

derived from real transcriptomes. The y-axis represents the number of the isoforms identified by different tools in different datasets. The color bars represent the known isoforms detected. The number in the bar represents the precision as indicated by the proportion of known isoforms among all identified isoforms. **(B)** Precision and recall plot depicting the performance of the assemblers (FLAIR, LAFITE and StringTie) on seven datasets when using RefSeq gene annotation.

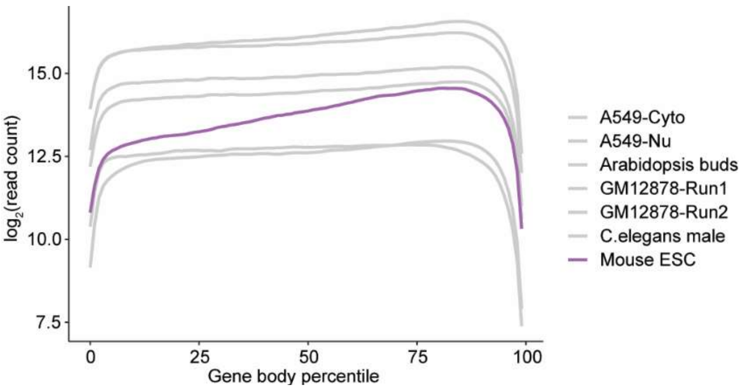

Figure S6. Gene body coverage of seven datasets used for performance evaluation. All samples showed comparatively even coverage except for Mouse ESC, indicating a potential degradation for the input RNA.

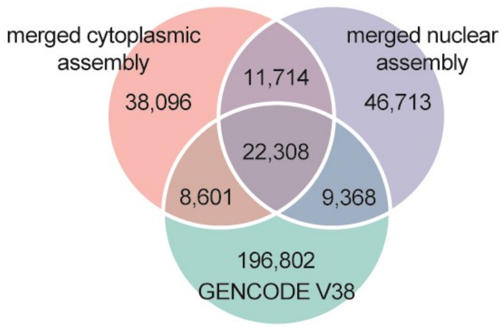

Figure S7. Venn diagram illustrating the transcript overlap among merged cytoplasmic assembly, merged nuclear assembly and GENCODE V38 annotation.

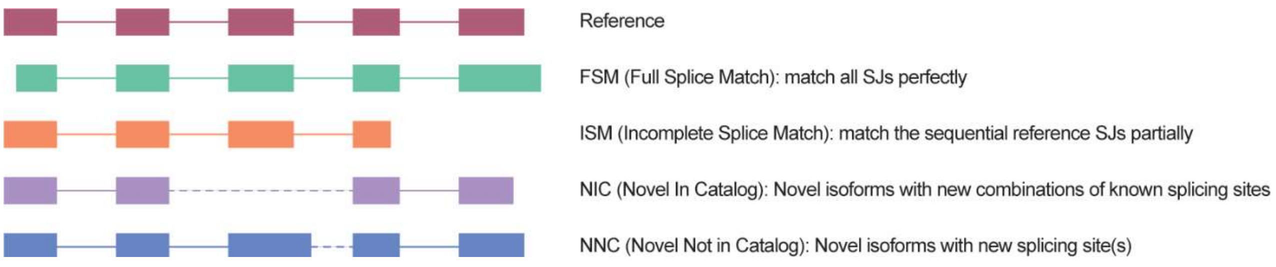

Figure S8. Schematic illustrates the classification of the isoforms based on their splicing structure similarity to reference annotation using SQANTI3. Novel splice junctions are indicated by dashed lines and known junctions by solid line. Isoforms are classified into five main categories: FSM, ISM, NIC, NNC, and other (*e.g.*, antisense, intergenic, or readthrough transcripts).

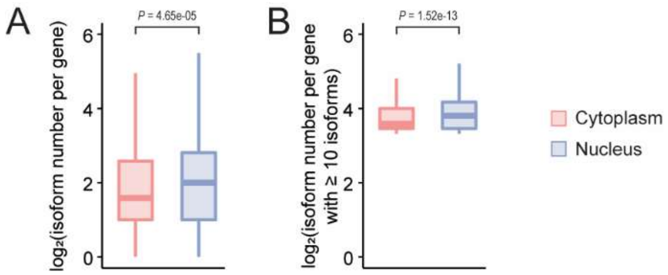

Figure S9. **(A)** Box plots showing the distribution of the isoform number per gene in the merged cytoplasmic and nuclear assembly. **(B)** Box plots showing the distribution of the isoform number per gene (with ≥ 10 isoforms) in the merged cytoplasmic and nuclear assembly. Only genes with detectable isoforms in both fractions were included in the analysis. *P*-values were determined by Mann-Whitney U test.

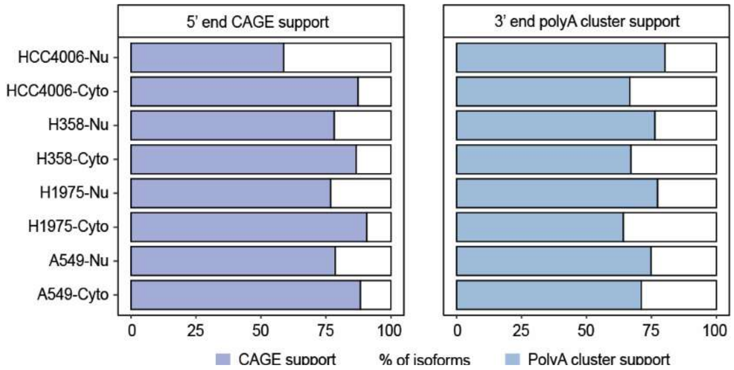

Figure S10. The proportion of the TSSs and TESs of the ISMs in each fraction assembly supported by CAGE peaks and the presence of a poly(A) clusters, respectively.

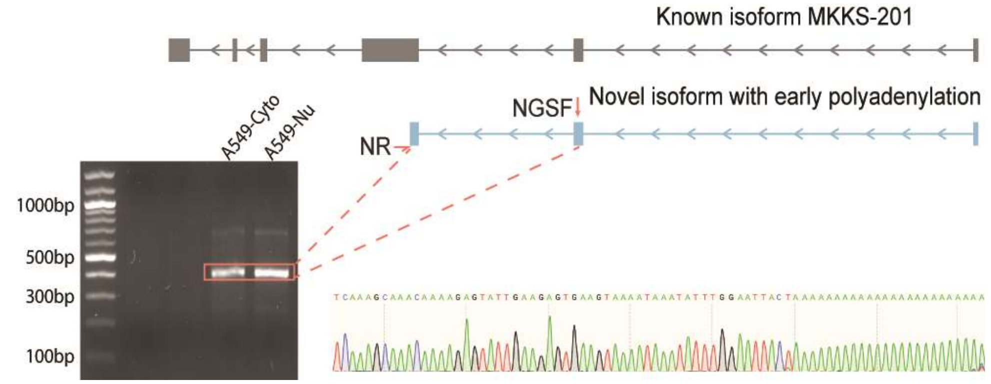

Figure S11. Result from 3′ RACE showing the early polyadenylation event detected in gene *MKKS*. NGSF: Forward gene specific primer for nested PCR; NR: reverse primer for nest PCR.

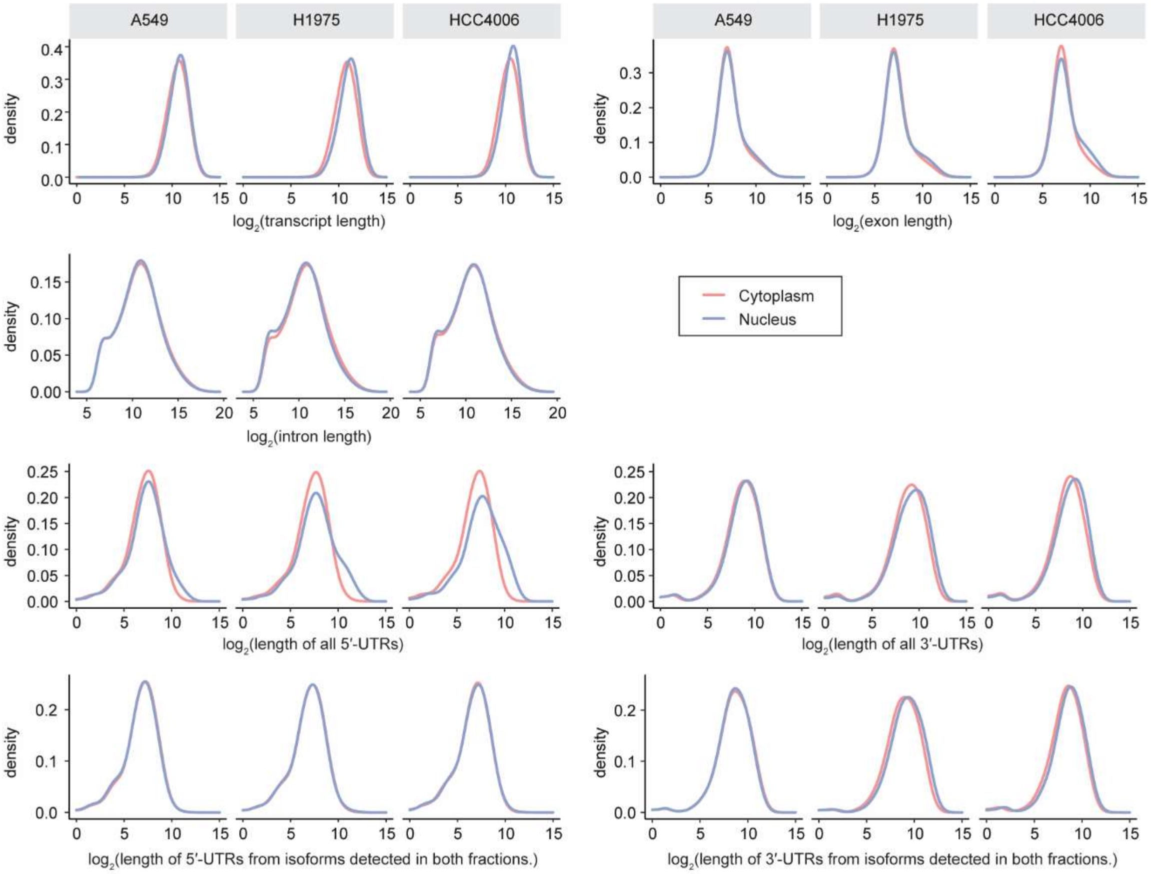

Figure S12. Density plots showing the length distribution of the transcript, exon, intron, 5′-UTR, and 3′-UTR detected in the cytoplasmic and nuclear fractions of A549, H1975, HCC4006.

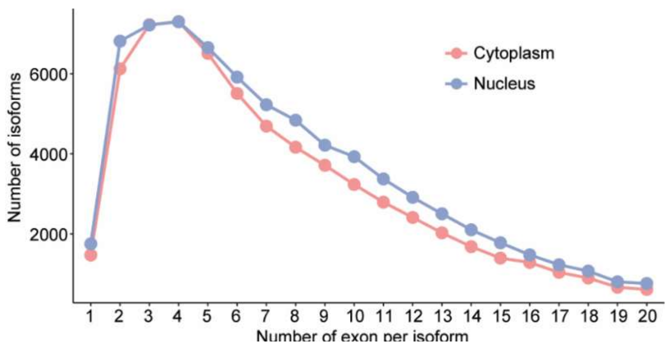

Figure S13. Distribution of the number of exons per isoforms. Comparatively more isoforms in nuclear fraction tend to have more exons (>=5).

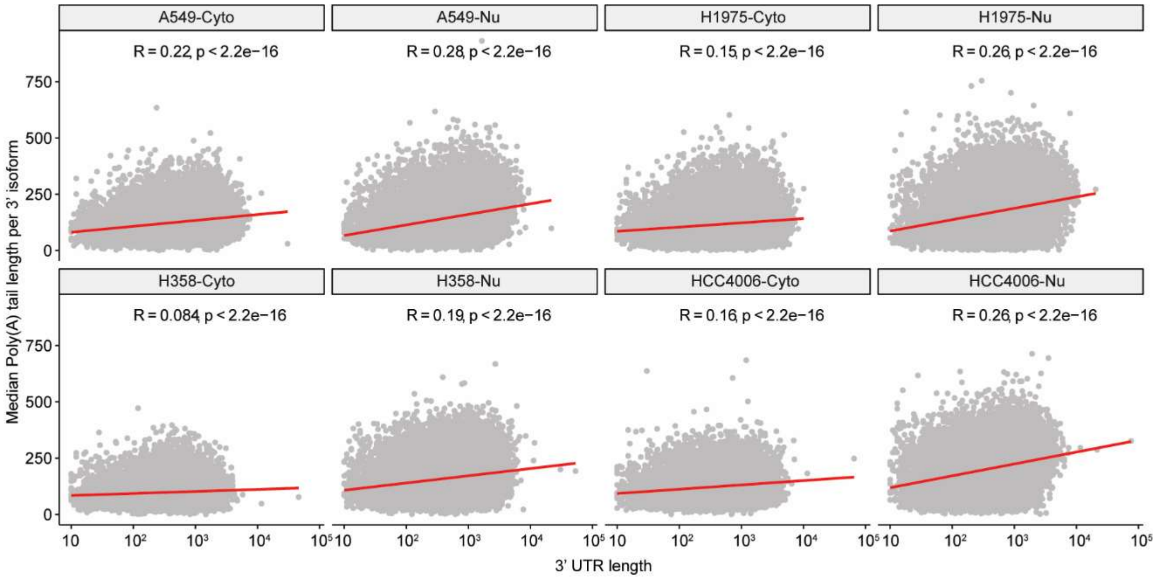

Figure S14. Pearson Correlation of 3'UTR length and median Poly (A) tail length detected in eight fractions.

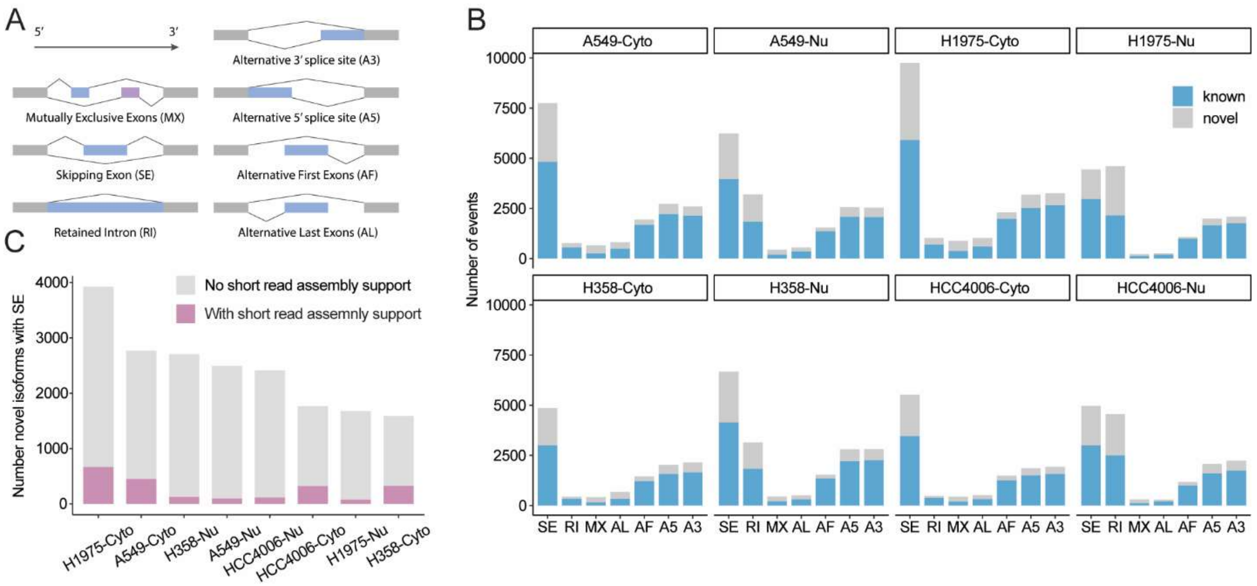

Figure S15. **Alternative splicing events in four cell lines.** (A) Schematic representation of different types of alternative splicing. A3: Alternative 3′ Splice Site; A5: Alternative 5′ Splice Site; AF: Alternative First Exon; AL: Alternative Last Exon; MX: Mutually Exclusive Exon; RI: Retained Intron; SE: Skipping Exon. (B) Stacked bar plot showing the number of known (blue bar) and novel (grey bar) splicing events detected for seven classes of alternative splicing events in each fraction. (C) Statistics of the short-read assembly support for the novel isoforms with SE event. Only a small partial of them can be reconstructed from short-read RNA-seq data (pink bar).

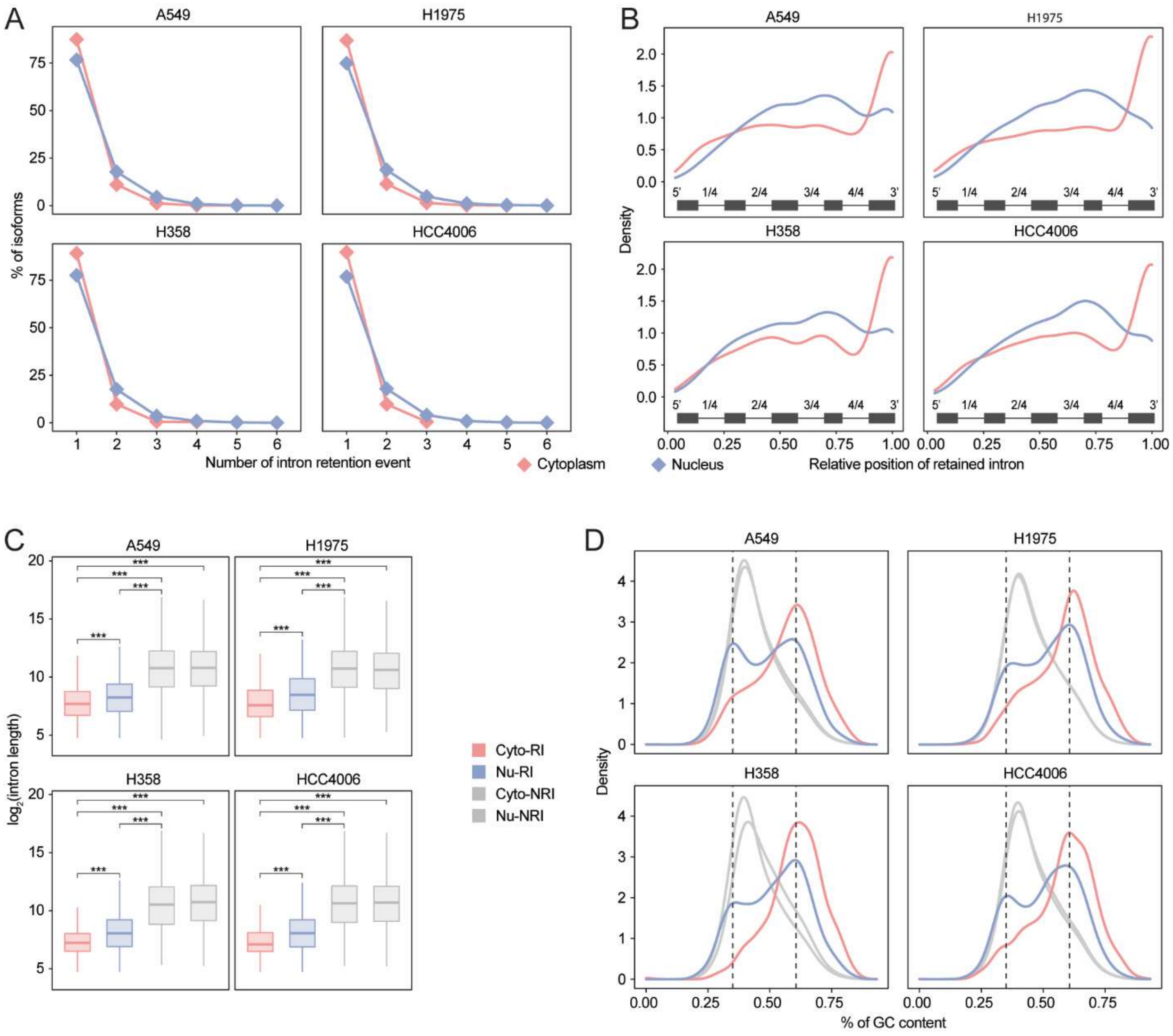

Figure S16. **Characterization of alternative splicing in four cell lines.** (**A**) Line plot showing the number of the RIs per isoform in the cytoplasmic and nuclear fractions, respectively. (**B**) Relative positions of RIs in relation to the other introns in the transcript structure, calculated by dividing the intron position by the total number of introns in the transcript. (**C**) Length distribution of the RIs detected in the cytoplasmic (Cyto-RI) and nuclear (Nu-RI) fractions and the non-retained introns (Cyto-NRI, Nu-NRI). **(D)** GC contents of the introns from different categories in **D**. (***P < 0.001, Mann-Whitney U test).

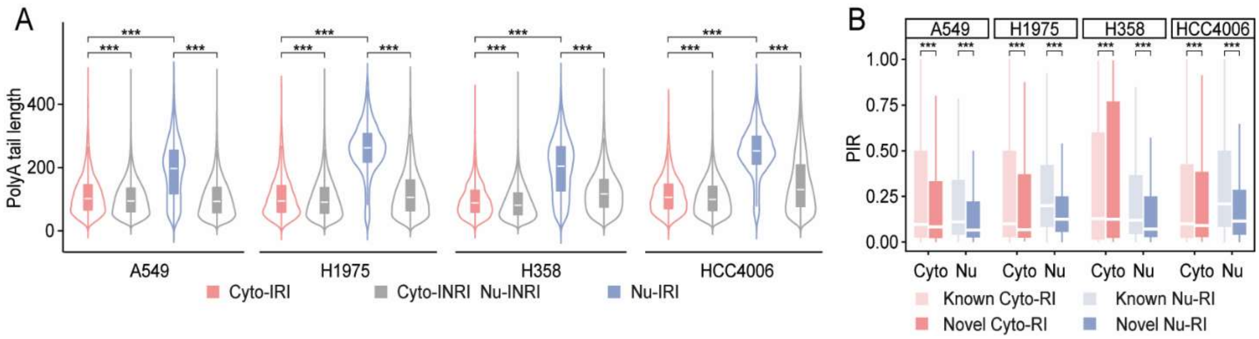

Figure S17. (A) Length distribution of poly(A) tail for the isoforms in the cytoplasmic and nuclear fractions with RIs (Cyto-IRI, Nu-IRI) as well as the rest isoforms without RIs (Cyto-INRI, Nu-INRI). **(B)** PIR values for the novel and known RI events detected in the cytoplasmic and nuclear fractions, respectively. (****P* < 0.001, Mann-Whitney U test)

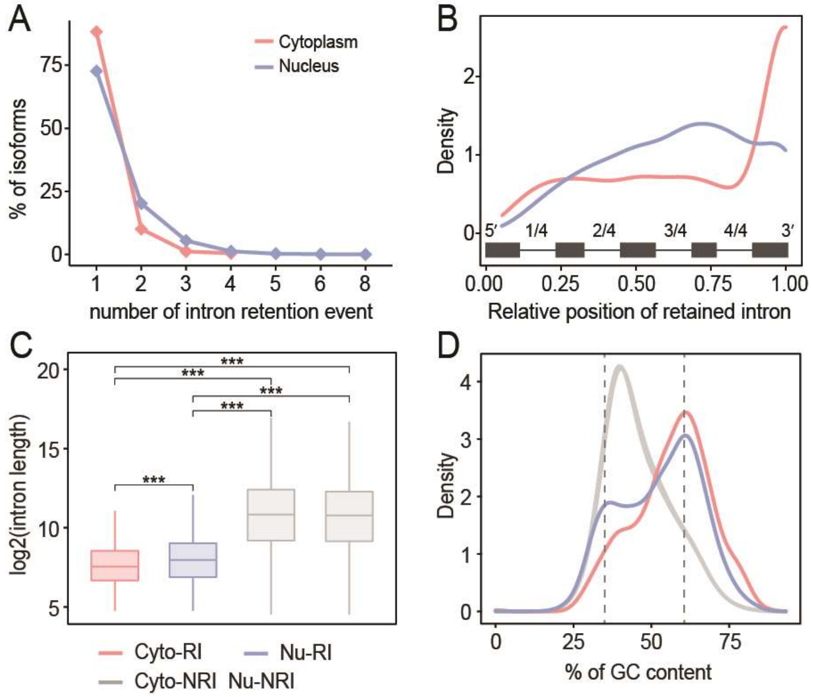

Figure S18. Characterization of the RI events (raw count >= 5) in subcellular fractions. (A) Line plot showing the number of the RI per isoform in the merged-cytoplasmic and merge-nuclear assembly, respectively. **(B)** Relative position of RI in relation to the other introns in the transcript structure, which is calculated by dividing the intron position by the total number of introns in a transcript. **(C)** Length distribution of the RIs detected in the cytoplasmic fraction (Cyto-RI) and nuclear fraction (Nu-RI) as well as the rest non-retained introns (Cyto-NRI, Nu-NRI). **(D)** Density of the GC content in the introns from different categories in D. (****P* < 0.001, Mann-Whitney U test)

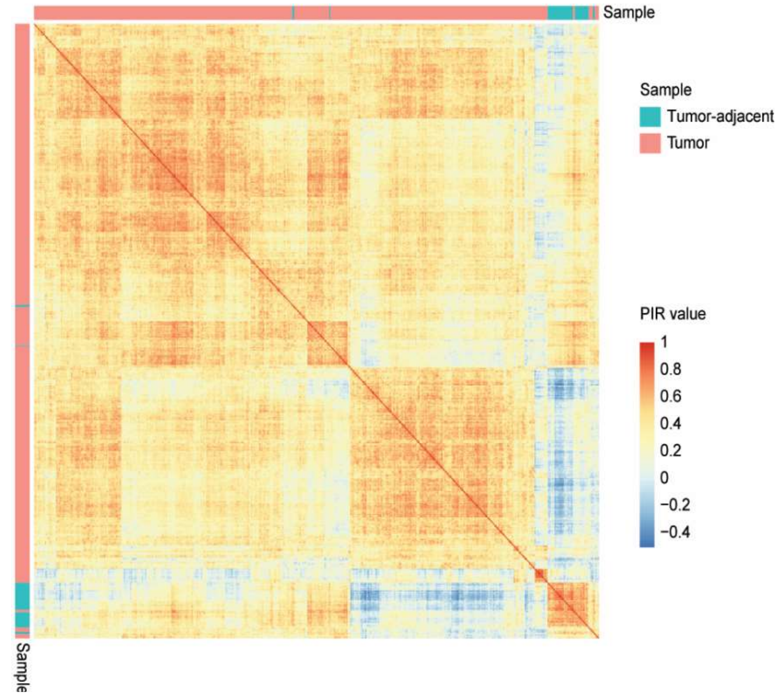

Figure S19. Hierarchical clustering of 596 samples from TCGA-LUAD cohort based on the PIR value of 255 differentially expressed RIs.

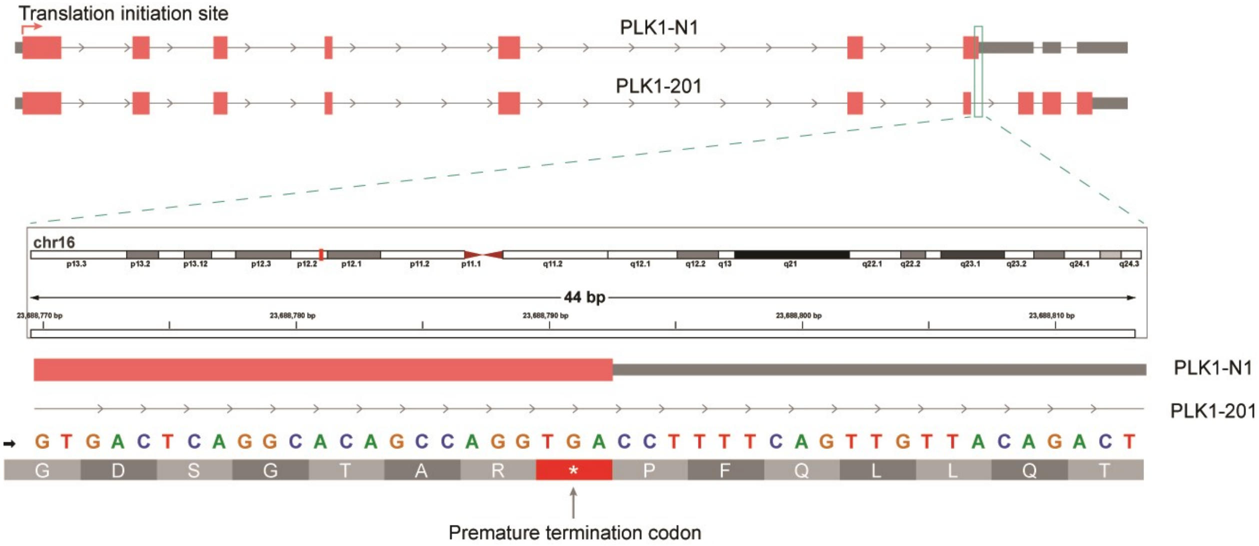

Figure S20. Genome browser view showing an in-frame premature termination codon contained in the novel retained intron in transcript *PLK1-N1*.

Table S1. Summary of the read statistics from in-house subcellular DRS datasets and public DRS datasets.

Sample	Raw read number	Filtered read number (Q>7)	Max read length	Average read length	Median read length	N50	N90
A549-Cyto	1,768,805	1,624,538	19,316	1,031	822	1,291	531
A549-Nu	2,531,889	2,343,511	14,906	1,056	904	1,332	573
H1975-Cyto	1,549,026	1,415,410	15,033	1,187	907	1,538	594
H1975-Nu	661,835	609,600	24,318	1,423	1,140	1,795	730
H358-Cyto	3,614,180	3,057,382	9,195	716	596	816	420
H358-Nu	1,480,459	1,371,413	16,013	1,303	1,079	1,641	683
HCC4006-Cyto	2,221,975	1,958,167	18,525	806	641	963	442
HCC4006-Nu	1,756,324	1,537,545	22,239	984	809	1,290	521
GM12878-Run1	830,753	612,732	38,475	780	577	1,247	470
GM12878-Run2	957,785	785,939	63,927	877	668	1,277	492

*GM12878-Run1 and GM12878-Run2 was retrieved from *Workman* et al. (*30*).

Table S2. Classification of the mouse ESC Assemblies generated from LAFITE, StringTie and FLAIR under the guide of reference annotation from GENCODE and RefSeq.

Class code	Description	GENCODE M28			RefSeq		
		LAFITE	StringTie	FLAIR	LAFITE	StringTie	FLAIR
=	complete match	6,539	5,372	3,657	3,727	3,576	2,998
c	contained in reference	83	11,118	12,214	103	10,437	12,515
e	single exon overlap reference intron	102	407	244	139	345	245
i	contained with a reference intron	299	649	438	504	824	629
j	multi-exon with at least one junction match	1,094	1,772	762	675	2,139	649
k	containment of reference	50	40	31	33	51	32
m	retained intron(s), not all introns matched	40	47	24	58	60	28
n	retained intron(s), all introns matched	164	221	211	89	275	227
o	overlap reference exon	203	1,756	537	130	871	382
p	possible polymerase run-on	267	457	384	211	353	289
u	intergenic	327	723	435	773	1,168	769
x	exonic overlap on opposite strand	81	137	108	92	135	129
y	contains a reference within its intron	2	1	0	3	4	0
total	total isoforms	9,251	22,700	19,045	6,537	20,238	18,892

Table S3. Summary of the functional Gene Ontology terms predicted by DeepFRI for the potential protein encoded by *AKT1-N1* .

Biological process	Gene Ontology term	DeepFRI score
organic substance metabolic process	GO:0071704	0.94
cellular metabolic process	GO:0044237	0.92
primary metabolic process	GO:0044238	0.9
nitrogen compound metabolic process	GO:0006807	0.86
macromolecule metabolic process	GO:0043170	0.78
cellular nitrogen compound metabolic process	GO:0034641	0.73
organonitrogen compound metabolic process	GO:1901564	0.7
cellular macromolecule metabolic process	GO:0044260	0.69
biosynthetic process	GO:0009058	0.63
organic substance biosynthetic process	GO:1901576	0.63
cellular biosynthetic process	GO:0044249	0.62
protein metabolic process	GO:0019538	0.58
cellular nitrogen compound biosynthetic process	GO:0044271	0.55
gene expression	GO:0010467	0.53

*DeepFRI score > 0.5 is considered statistically significant.

Table S4. Summary of the identified m6A modifications.

	A549-Cyto	A549-Nu	H1975-Cyto	H1975-Nu	H358-Cyto	H358-Nu	HCC4006-Cyto	HCC4006-Nu	Merged-Cyto	Merged-Nu
No. of m6A sites on non-coding isoform	973	948	1,712	399	1,485	901	906	1,183	2,307	2,868
No. of non-coding isoforms with m6A	215	202	184	77	360	191	220	259	553	578
No. of m6A sites on coding isoform	17,994	17,379	39,358	5,013	14,934	22,237	12,474	5,698	30,435	28,991
No. of coding isoforms with m6A	2,818	2,481	3,121	995	2,291	2,938	2,209	1,087	4,284	3,780
Total No. of m6A sites	18,967	18,327	41,070	5,412	16,419	23,138	13,380	6,881	32,742	3,1859
Total No. of isoforms with m6A	3,033	2,683	3,305	1,072	2,651	3,129	2,429	1,346	4,837	4,358

Table S5. Summary of the alternative splicing events detected in each DRS dataset.

	A549-Cyto	A549-Nu	H1975-Cyto	H1975-Nu	H358-Cyto	H358-Nu	HCC4006-Cyto	HCC4006-Nu
A3 (alternative 3′-acceptor)	2,597	2,530	3,250	2,082	2,139	2,807	1,924	2,225
A5 (alternative 5′-donor)	2,725	2,559	3,176	1,988	2,020	2,797	1,858	2,073
AF (alternative first exon)	4,130	3,403	5,387	2,134	2,673	3,278	2,803	2,557
AL (alternative last exon)	1,340	982	1,687	489	1,100	863	807	656
MX (mutually exclusive exon)	656	440	883	221	417	448	440	307
RI (intron retention)	796	3,365	1,070	4,863	452	3,336	487	5,071
SE (exon skipping)	7,743	6,232	9,738	4,436	4,856	6,667	5,513	4,963

Table S6. Summary of the read statistics based on the Illumina RNA-seq data from eight fractions.

Sample	Total read pairs	Clean read pairs	Uniquely mapped read pairs
A549-Cyto	63,475,158	62,078,740	58,743,577
A549-Nu	65,751,528	62,536,122	60,043,996
H1975-Cyto	63,881,969	62,563,458	59,702,829
H1975-Nu	63,806,958	49,690,663	46,920,398
H358-Cyto	60,857,877	56,748,204	47,041,804
H358-Nu	74,221,705	67,756,964	63,994,345
HCC4006-Cyto	68,116,027	66,468,905	60,279,916
HCC4006-Nu	61,446,375	58,943,733	55,895,805
